# Human–Robot Interaction in Indoor Mobile Robotics: Current State, Interaction Modalities, Applications, and Future Challenges

**DOI:** 10.3390/s26061840

**Published:** 2026-03-14

**Authors:** Arman Ahmed Khan, Kerstin Thurow

**Affiliations:** Center for Life Science Automation, University of Rostock, F.-Barnewitz-Str. 8, 18119 Rostock, Germany

**Keywords:** human–robot interaction, indoor mobile robots, interaction modalities, multimodal interfaces, usability and trust, safety, privacy, regulatory considerations

## Abstract

This paper provides a comprehensive survey of Human–Robot Interaction (HRI) for indoor mobile robots operating in human-centered environments such as hospitals, laboratories, offices, and homes. We review interaction modalities—including speech, gesture, touch, visual, and multimodal interfaces—and examine key user experience factors such as usability, trust, and social acceptance. Implementation challenges are discussed, encompassing safety, privacy, and regulatory considerations. Representative case studies, including healthcare and domestic platforms, highlight design trade-offs and integration lessons. We identify critical technical challenges, including robust perception, reliable multimodal fusion, navigation in dynamic spaces, and constraints on computation and power. Finally, we outline future directions, including embodied AI, adaptive context-aware interactions, and standards for safety and data protection. This survey aims to guide the development of indoor mobile robots capable of collaborating with humans naturally, safely, and effectively.

## 1. Introduction

The integration of mobile robots into indoor environments marks a significant paradigm shift in robotics—from isolated industrial applications to direct collaboration with humans in shared spaces [1]. Indoor mobile robots are increasingly deployed across diverse environments, including hospitals [2], laboratories [3], offices, and residential settings [4]. This shift requires Human–Robot Interaction (HRI) capabilities to be sophisticated to enable natural, safe, and efficient collaboration between humans and robotic systems.

Several converging factors motivate the development of effective HRI in indoor mobile robotics.

**Aging population:** An aging global population creates a critical need for assistance. By 2050, the world’s population of people aged 60 years and older will double to 2.1 billion; 1 in 6 people worldwide will be over 65 by 2050, which is a significant increase from 1 in 11 in 2019, according to the World Health Organization [5]. This aging population and growing healthcare needs require the deployment of robots in hospitals, care facilities, and home care. Robots can relieve caregivers by performing routine tasks, thereby increasing the efficiency of patient care [6]. The use of mobile robots enhances and enables independent living for the elderly and those with disabilities. The use of smart wheelchairs with Human–Robot Interaction via artificial intelligence increases usability, learnability, efficiency, satisfaction, and a sense of independence and dignity among elderly and mobility-impaired individuals [7,8]. Matthias and Markus proposed an intelligent wheelchair that can navigate in indoor environments and accompany any person. In addition, it allows social interaction while walking to relieve relatives or nursing staff, who otherwise need to push the wheelchair [9]. Shannon Vallor in her paper “Carebots and Caregivers” introduced the concept of “carebots” and argued that the ethical evaluation of these systems must extend beyond the impact on patients to consider the moral value of caregiving practices for the caregivers themselves, examining whether robots sustain or deprive them of the internal goods of caring [10]. The TORNADO cloud-integrated robotics platform, featuring people-aware navigation and dexterous manipulation, includes a validation scenario specifically focused on patient support in a hospital palliative ward [11]. This ageing population requires robots to assist, care, help, and socially interact. To achieve that goal, we need the human-centric design of Human–Robot Interaction.

**Labor shortage:** Another major motivating factor to develop robots with intelligent and user-accepted Human–Robot Interaction is due to the labor shortage. To have the robot cooperate and coordinate with humans, it needs to understand human norms and proxemics. Cao and Tam proposed a search and fetch operation for the mobile manipulation robots with a multimodal Human–Robot Interaction via gesture, voice, and face recognition. This can address the persistent labor shortage in roles that require complex, repetitive tasks in indoor environments like manufacturing factories [12]. Beyond simple repetition, the economic viability of automation is increasingly driven by the modernization of existing “brownfield” facilities. Unlike “greenfield” projects built from scratch, brownfield environments require Autonomous Mobile Robots (AMRs) that can navigate without fixed infrastructure like rails, allowing companies to automate gradually without expensive shutdowns or remodeling [13]. In labor-intensive industries like textile composite production, where full automation is cost-prohibitive, collaborative robots (cobots) offer a middle ground. They reduce production time and costs while preserving the tacit knowledge of skilled human experts who are becoming scarcer due to aging workforces [14]. This trend extends to agriculture, where robotic planters are being designed to remedy future farmer shortages by optimizing energy usage and draft force [15]. One of the common things among these users is their lack of technical skills and training to operate and interact with robots, and hence, the human-centric design of interaction becomes paramount. In the service and healthcare sectors, the integration of Generative AI (GenAI) which include large language models (LLMs), large behavioral models (LBMs), and agentic AI is transforming the economic landscape by enabling “citizen developers”—frontline employees who can train and fine-tune robots without coding skills, leading to cost-effective service excellence [16]. Real-world applications demonstrate significant returns on investment; for instance, the deployment of Moxi robots has saved clinical staff over 575,000 h and 1.5 billion steps, directly addressing clinician burnout and operational inefficiency [17]. Furthermore, humanoid robots are being explored as a solution to hospital labor shortages, capable of performing teleoperated clinical tasks with human-like dexterity [18]. The deployment of mobile robots in healthcare settings has demonstrated promising results in improving operational efficiency and reducing the workload on medical staff [19,20]. Service robots have evolved from simple delivery systems to sophisticated platforms capable of complex Human–Robot Interaction [21,22].

**Pandemic Response and Biosecurity:** The COVID-19 pandemic acted as a catalyst for robotic adoption, highlighting the advantage of systems with intrinsic immunity to pathogens [23]. Robots became essential for maintaining “social distancing” in medical settings. For example, the “Dr. Spot” quadruped robot was developed to measure vital signs (skin temperature, heart rate, SpO2) without direct contact, preserving Personal Protective Equipment (PPE) and protecting healthcare workers [24]. Mobile robots are assessed for deployment in isolation-room hospital settings to execute tasks like remote supply delivery and medication distribution, aiming to minimize the risk of cross-contamination and reduce staff workload, particularly during infectious disease outbreaks like the COVID-19 pandemic [25,26]. In extreme cases, such as in Wuhan, China, entire wards were temporarily run by robots to deliver food and medicine to quarantine patients, minimizing human exposure [27]. Telepresence robots also gained traction, allowing isolated patients to interact with families and doctors while avoiding contagion risks [28]. Beyond direct care, robots have been utilized for disinfection, logistics, and waste handling, effectively breaking the chain of virus transmission [26]. This protection extends to surgical oncology, where robotic systems allow for precise interventions while adhering to strict safety protocols to prevent viral aerosolization [29]. Therefore, whenever a human is involved, which is very likely the case in the indoor environments of hospitals and laboratories, the robot must have a Human–Robot Interaction model covering the basics like human-aware navigation, intuitive interaction, and likable presence.

**Occupational and Psychological Safety:** Beyond biological hazards, HRI addresses physical and mental safety in industry. In the construction sector, which faces high accident rates, machine learning models are being used to predict human trust in robots based on physiological data (such as skin temperature), ensuring that human–robot collaboration (HRC) remains safe and productive [30]. However, safety is also psychological. The isolation caused by pandemics or varying abilities affects mental health. Social robots with “hybrid face” designs have been deployed to support the mental health of older adults and those with dementia, providing companionship and “human-like” conversation to mitigate isolation [31]. Research in aged care suggests that while robots can relieve the physical workload, their acceptance depends heavily on their reliability and the emotional reactions of the staff, emphasizing that psychological safety is a prerequisite for successful implementation [32]. Ultimately, robots are tasked with the “dull, dirty, and dangerous” jobs—from welding to patient lifting—enhancing the overall safety profile of modern work environments [33].

**Figure 1:**
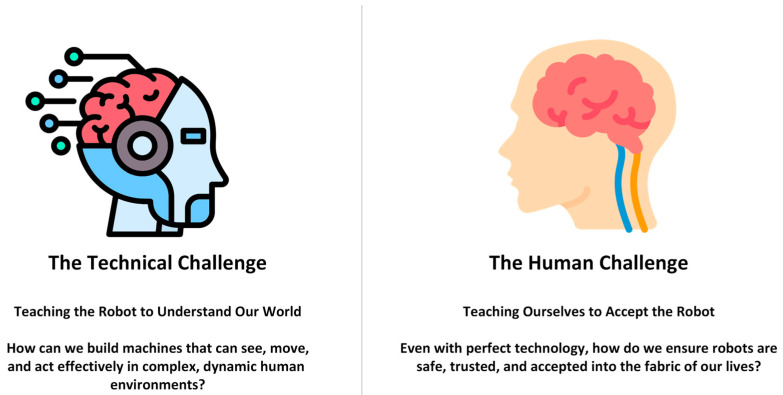
Twofold challenge of Indoor Human–Robot Interaction.

The challenge for Human–Robot Interaction in indoor environments is twofold. One is the technical challenge, and the other is the human challenge (Figure 1).

- **Complexity of environments:** Indoor environments such as hospitals, laboratories, and offices are dynamic, tightly structured, and populated with many moving obstacles. Robots must not only navigate but also consider human activities and interact safely. Effective HRI supports adaptive, context-sensitive, flexible collaboration [34].
- **Extending robot functionality:** Natural interaction methods such as speech, gestures, touch, or gaze enable robots to perform more complex tasks that would be difficult to manage without HRI. These capabilities make robots more intuitive and “human-like” to operate, lower the learning barrier for users, and improve task efficiency.
- **User acceptance and trust:** The success of robotic systems depends heavily on user trust and acceptance. Robots must behave in a comprehensible, reliable, and socially appropriate manner to ensure that users can rely on them and use them effectively in daily life [35]. Trust is a prerequisite for user acceptance [35,36,37]. It depends heavily on crucial human-centric factors, such as whether users are willing to accept the technology and trust it to perform its tasks reliably [38]. Only then can we have the long-term adaptation of mobile robotics in our personal spaces.
- **Safety and ethical considerations:** In human-centered environments, safety is paramount. HRI contributes to predictable, transparent, and ethically sound interactions, for example, through clear communication, privacy protection, and the respectful handling of human autonomy.

**Figure 2:**
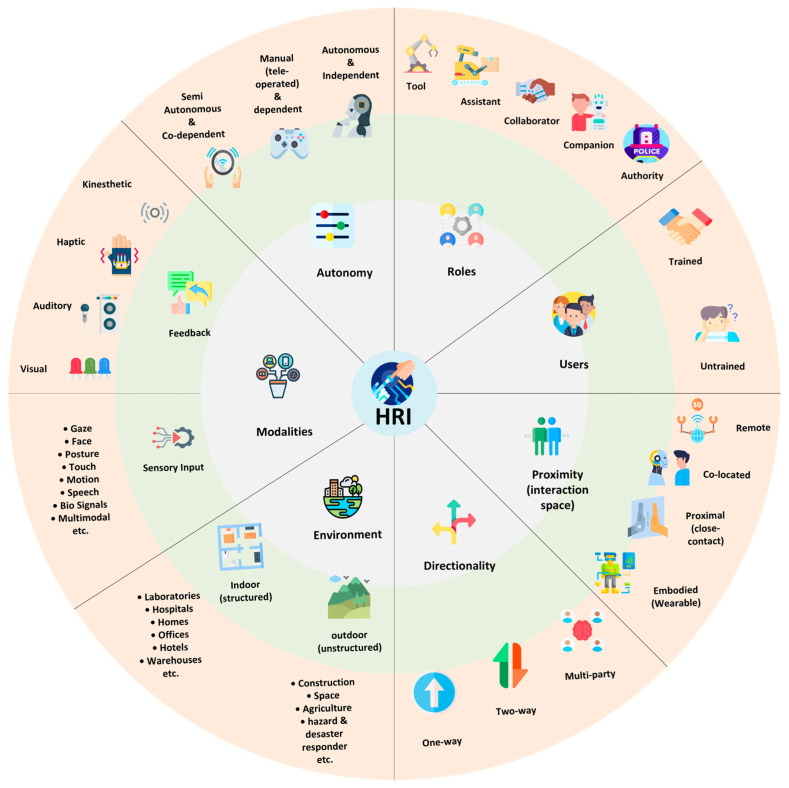
HRI classifications.

HRI can be classified along multiple axes; Goodrich and Schultz survey HRI in terms of robot autonomy, interaction roles, and team organization [1]. Parasuraman et al. formalize levels of automation for information acquisition, analysis, decision, and action—a framework widely adopted in HRI design and evaluation [39]. Steinfeld and Fong talked about common matrices for Human–Robot Interactions [40]. Bartneck et al. covered measurement instruments for the anthropomorphism, animacy, likeability, perceived intelligence, and the perceived safety of robots [41]. Figure 2 represents the different dimensions of HRI classification.

This review provides a comprehensive and integrative synthesis Human–Robot Interaction (HRI) in indoor mobile robotics. Unlike prior surveys that focus on isolated subdomains, we systematically combine three perspectives that are often treated separately: (i) technical aspect of HRI, including modalities such as speech, gesture, touch, visual, and emerging LLM-enabled interfaces; (ii) human aspect of HRI, encompassing usability, trust formation, social acceptance, and long-term user experience; and (iii) practical and regulatory consideration, including safety engineering, privacy considerations, deployment constraints, and the compliance framework.

By bridging fragmented literature across robotics, human–computer interaction, healthcare technology, and safety engineering, this review addresses a critical gap: the lack of a unified 2025 perspective that connects technical implementation with socio-technical integration in real deployment contexts. We analyze representative case studies from operational systems to ground theoretical insights in practice and to derive transferable design principles.

In addition to synthesizing the state of the art, we identify key open research challenges, including the need for longitudinal studies on trust dynamics, the cross-cultural validation of HRI models, scalable economic deployment frameworks, and standardized interoperability architectures for heterogeneous robot fleets. Addressing these challenges is essential for advancing indoor mobile robotics toward natural, safe, economically sustainable, and socially accepted human–robot collaboration.

Finally, we analyze representative case studies and examine current approaches with highlights of future research directions aimed at advancing the field toward more natural, safe, and effective human–robot collaboration in indoor environments.

## 2. Review Methodology

To ensure a rigorous and comprehensive analysis of the current state of Human–Robot Interaction (HRI) in indoor environments, this survey follows a systematic literature review approach. This methodology was designed to minimize selection bias and ensure the inclusion of both foundational theories and the most recent high-impact developments, particularly in the rapidly evolving fields of Large Language Models (LLMs) and embodied AI.

### 2.1. Search Strategy and Data Sources

A systematic search was conducted across two primary scientific databases: Scopus and Google Scholar. To address the interdisciplinary nature of HRI, medical-focused databases (e.g., PubMed) were also consulted specifically for applications in healthcare robotics. For Google Scholar, only the first 200 results sorted by relevance were screened to ensure feasibility and quality control, due to the lack of structured filtering mechanisms comparable to curated academic databases.

The search strategy employed a combination of keywords using Boolean operators, adapted for each database’s syntax. The core search strings included the following:

- **Primary Descriptors:** (“Mobile Robot” OR “Service Robot” OR “Social Robot” OR “Embodied AI”)
- **Context Qualifiers:** AND (“Indoor” OR “Hospital” OR “Home” OR “Office” OR “Laboratory”)
- **Interaction Descriptors:** AND (“HRI” OR “Human-Robot Interaction” OR “Trust” OR “Social Acceptance” OR “Social Navigation”)
- **Emerging Technologies (2023–2025):** Specific supplementary searches were conducted for (“Large Language Models” OR “LLM” OR “Generative AI” OR “Vision-Language Models”) AND “Robotics” to address the paradigm shift identified by recent scholarship.

### 2.2. Inclusion and Exclusion Criteria

The initial search yielded over 1724 potential records from the searched platforms. Duplicate records were removed prior to screening. The remaining articles were screened first by title and abstract and subsequently by full-text review based on the following criteria:

#### 2.2.1. Inclusion Criteria

- Platform: Studies focusing on mobile robotic platforms (wheeled only) operating in indoor environments.
- Focus: Papers where HRI, user experience (UX), or human-aware navigation were a primary variable.
- Recency: Priority was given to works published between 2015 and 2025, with exceptions made for seminal foundational papers that established core HRI metrics.
- Language: Peer-reviewed articles and high-impact conference proceedings in English.
- Domain Expertise: When meeting the inclusion criteria, peer-reviewed contributions from the authors’ group were included to ensure completeness within the life-science robotics domain. These papers were subjected to the same screening criteria.

#### 2.2.2. Exclusion Criteria

- Environment: Outdoor robotics (e.g., autonomous driving, agriculture) and underwater/aerial drones were excluded.
- Morphology: Stationary industrial manipulators (robotic arms) operating in caged environments, as well as any legged robots, were excluded, except when mounted on mobile bases (mobile manipulation). This restriction ensures comparability in navigation constraints and interaction paradigms, as legged robots introduce fundamentally different locomotion and perception dynamics.
- Methodology: Simulation-only studies that lacked human validation or real-world applicability were generally excluded, except where they demonstrated novel theoretical frameworks.

### 2.3. Dataset Composition

**Figure 3:**
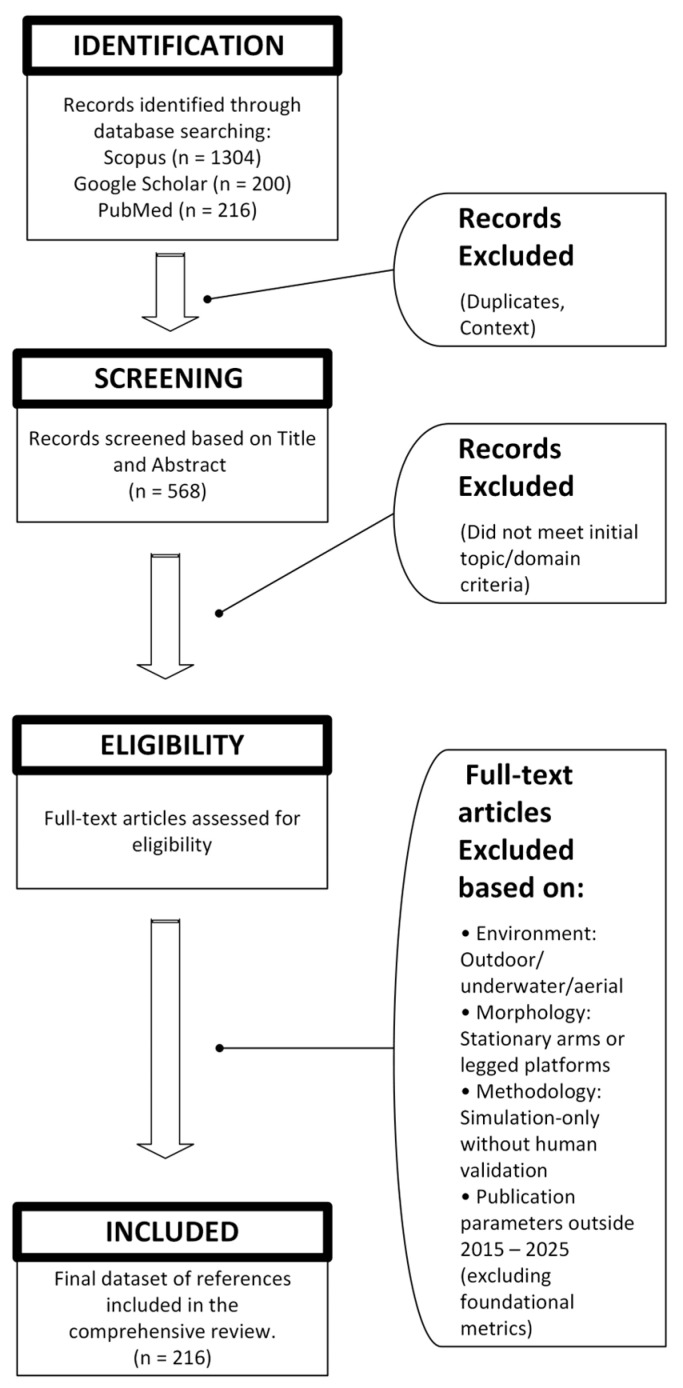
Flow diagram of the literature selection process.

The final dataset consists of 216 references. To ensure that the review remains timely for a 2025 audience, the bibliography is heavily weighted towards recent advancements, with a significant proportion of citations from the 2020–2025 period. This distribution captures the post-COVID-19 acceleration in service robotics and the integration of generative AI. The Preferred Reporting Items for Systematic reviews and Meta-Analyses (PRISMA) can be seen in Figure 3. 

### 2.4. Case Study Selection

To bridge the gap between academic theory and practical deployment, three commercial robotic platforms—Moxi (Diligent Robotics), Temi, and Amazon Astro—were selected for the detailed analysis in Section 6. These specific platforms were chosen based on the following:

1. **Market Maturity:** All three have moved beyond the prototype stage to widespread commercial deployment.
2. **Domain Representation:** They represent three distinct indoor domains analyzed in this review: Healthcare (Moxi), Telepresence/Office (Temi), and Domestic/Home (Astro).
3. **Interaction Diversity**: They exhibit a range of interaction modalities, from multimodal mobile manipulation to consumer-grade social presence, allowing for a comparative analysis of trust and acceptance metrics.

The selection is not intended to be exhaustive but illustrative. The three platforms were chosen to represent distinct deployment domains and interaction paradigms rather than market dominance or technological superiority. This ensures analytical comparability while acknowledging the broader ecosystem of indoor mobile service robots. Alternative platforms were screened but excluded due to limited deployment data or insufficient publicly available interaction metrics.

### 2.5. Reproducibility Trends: A Crisis and a Shift

HRI has historically suffered from a “reproducibility crisis” because user studies often rely on custom hardware, specific physical environments, and subjective surveys that are hard to replicate [42].

- The “Context” Problem: Unlike pure code benchmarks (like in computer vision), HRI results depend heavily on the physical context (e.g., a hospital hallway vs. a lab) and social context (e.g., cultural norms of the participants). Papers now increasingly demand “conceptual replication” (testing the same hypothesis in a new context) rather than just exact replication [43].
- Standardized Scenarios: There is a trend toward using standardized task boards and scripted interaction protocols (e.g., the “socnav” benchmarks [44]) so different labs can test robots on the exact same social navigation scenarios.

### 2.6. Benchmarking Standards

Currently, no single “gold standard” exists, but the community is coalescing around specific metrics for Social Navigation and Interaction Quality.

#### 2.6.1. Social Navigation Metrics (Quantitative)

- For mobile robots moving among people, “success” is not just reaching the goal; it is how you get there.
- Safety: Minimum distance to human, collision rate.
- Comfort/Social Norms:
  - Social Work: Number of intrusions into “personal space” (proxemics violations).
  - Jerky Motion: Path irregularity or acceleration changes (smoothness), which correlates with human trust.
- Efficiency: Success weighted by path length is the standard metric. It penalizes robots that take safe but absurdly long detours.

#### 2.6.2. Interaction and Trust Metrics (Qualitative/Subjective)

Since “feeling safe” is subjective, standardized questionnaires are critical for comparability.

- Godspeed Questionnaire: The most widely used standard for measuring Anthropomorphism, Animacy, Likeability, Perceived Intelligence, and Perceived Safety [41].
- Trust in Automation Scales: Specific scales (like the Jian et al. Trust Scale) are now standard for measuring how much a user trusts a mobile robot [45].
- NASA-TLX: Standard for measuring cognitive load—i.e., how mentally demanding it was for a human to interact with the robot [46].
- SUS for Perceived Usability: The System Usability Scale is standardized questionnaire for the assessment of perceived usability [47].

#### 2.6.3. Datasets and Simulators

- SEAN 2.0 (Social Environment for Autonomous Navigation): A popular simulation platform for benchmarking social navigation algorithms [48].
- SocNavBench: A dataset/benchmark specifically for measuring how well robots predict and move around human social behaviors [44].
- Scand: A large-scale dataset of socially compliant navigation demonstrations [49].
- RISE: It is a tool to generate human robot interaction scenarios [50].

## 3. Applications of Indoor Mobile Robots: Cross-Domains and Domain-Specific Challenges

Indoor mobile robots are autonomous or semi-autonomous robotic systems that operate across diverse indoor environments, such as hospitals, laboratories, offices, industrial facilities, and private homes. These environments differ in structure, predictability, and user characteristics. As summarized in Table 1, indoor operational context can be broadly classified as structured and predictable or unstructured and dynamic. This distinction provides a useful framework for understanding both cross-domain and domain specific requirements.

Indoor mobile robots perform tasks ranging from transportation and assistance to monitoring and social interaction while interacting safely and effectively with humans and dynamic surroundings. Compared to outdoor industrial robots, they operate in fundamentally different contexts, which impose specialized capabilities and design considerations [51]. These environments present unique constraints that directly influence interaction design and user experience. Indoor mobile robots operate in constrained, dynamic environments where they must navigate around humans, devices, furniture, and other obstacles while maintaining social appropriateness [52]. The interaction design must accommodate diverse user groups with varying technical expertise, from healthcare professionals to elderly residents [53] as depicted in Table 2.

### 3.1. Cross-Domain HRI Challenges

Regardless of the environment, several challenges are shared across domains:

- **Safe Navigation in Human-Populated Spaces:** Robots must operate in constrained, dynamic environments with humans, furniture, and moving obstacles. They need precise collision avoidance while maintaining efficiency and must adapt to unpredictable human behavior [34,54]. Structured environments, like laboratories, emphasize exact path following, whereas homes or healthcare settings require dynamic adaptation to moving people and clutter [55,56].
- **Social Appropriateness and Intent Communication:** Across domains, robots must behave in socially intelligible ways. Mechanisms include visual signaling, auditory cues, gesture-based communication, and projected indicators of movement or intent [57,58]. Such communication increases the predictability and legibility of robot motion, thereby enhancing human–robot trust, whether in office corridors, hospital hallways, or shared home spaces [59,60].
- **Heterogenous User Groups:** Indoor robots interact with highly trained professionals, administrative staff, elderly residents, children, or casual visitors [6,61]. Each group has different expectations, technical literacy, and cognitive loads [62]. The interaction design must accommodate these differences, providing clear, context-appropriate feedback without overloading users, accounting for varying levels of psychosocial functioning [63].
- **Infrastructure Integration:** Robots must interface with existing buildings and workflow infrastructure, including elevators, automatic doors, IT networks, and laboratory management systems [64,65]. Reliable integration ensures smooth operation and minimal disruption, often requiring standardized “plug and play” frameworks for seamless deployment [19,66].
- **Trust, Privacy, and Ethical Considerations:** Especially in healthcare and residential settings, robots often process sensitive data [67]. The cross-domain HRI design must safeguard privacy, implement consent mechanisms, and maintain transparency to ensure user trust and avoid the ethical pitfalls related to surveillance or autonomy [35,68,69].

### 3.2. Domain-Specific Requirements

While indoor mobile robots share certain cross-domain challenges such as safe navigation, clear communication of intent, infrastructure integration, and accommodation of heterogeneous user groups, the relative importance and technical implementation of these challenges vary significantly by domain. Each environment imposes unique operational constraints, interaction priorities, and user expectations, which shape both the design of the robot and the nature of Human–Robot Interaction.

#### 3.2.1. Healthcare and Elderly Care

Education and healthcare are particularly fertile application areas for domain-specific Human–Robot Interaction [61,70]. Healthcare facilities represent one of the most demanding indoor environments due to sterility requirements, strict patient privacy constraints, time-critical workflows, and the emotional sensitivity of users. Robots such as Moxi (Diligent Robotics) assist nursing staff with routine tasks, including delivering supplies or transporting medications [71]. These assistance tasks involve more direct support to users and often require an understanding of individual user preferences, routines, and intentions. Such applications rely on advanced interaction capabilities, including speech recognition, gesture interpretation, contextual understanding, and long-term user modeling to personalize the robot’s behavior and responses over time.

Common applications in healthcare settings include medication reminders for elderly patients, health monitoring and data collection, or educational activities in classrooms and laboratories [72]. Robots in these environments must navigate precisely around medical equipment, patient beds, and healthcare staff, while strictly maintaining sterility and safety protocols [73]. Platforms such as “Marvin”, an omni-directional assistant for domestic environments, are designed to support elderly monitoring and remote presence [74]. Additionally, robots must recognize and respond appropriately to human presence, avoid disrupting workflows, and always ensure patient privacy.

In residential care, robots increasingly provide emotional support and assistance with activities of daily living for elderly individuals [22]. The deployment of heterogeneous mobile robot fleets in hospitals, such as Tartu University Hospital, demonstrate the practical impact of these systems: robots perform time-critical object transportation tasks, moving samples from intensive care units (ICUs) to hospital laboratories through crowded and narrow hallways [75]. The development of usable autonomous mobile robots requires careful consideration of user needs, environmental constraints, and task-specific requirements [21]. Social robots in hospitals play roles ranging from patient companionship to healthcare delivery. They generally yield high user satisfaction when they have multimodal communication and personalized behaviors to mitigate occasional user fear or frustration [76,77].

#### 3.2.2. Laboratory Environments

Laboratory environments introduce considerable complexity due to the presence of expensive and sensitive equipment, hazardous chemicals, and strict procedural requirements [3]. Robots operating in laboratories must handle delicate instruments accurately, maintain contamination control, navigate safely around staff and equipment, and adapt to dynamic experimental setups [64]. Integration with laboratory management systems, adherence to precise spatial paths, and responsiveness to dynamic workspace changes are essential to ensure both safety and workflow efficiency.

Multi-floor labware transportation systems such as the MOLAR Automated Guided Vehicles (AGVs) developed at the Center for Life Science Automation (CELISCA) execute complex workflows, moving labware and materials between workbenches located on different floors [78,79]. These robots are integrated with laboratory infrastructure, including automatic doors and elevators opening [64,78,79]. Research also addresses methods to correct unexpected localization errors to maintain operational safety [80]. Similarly, the H2O robot at CELISCA uses StarGazer sensors to navigate via ceiling landmarks and employs hybrid elevator-controlling strategies that combine robot arm manipulation and wireless control [65,79]. For both systems, social navigation and interaction with humans are critical HRI components.

The mobile robot Kevin was specifically designed to handle the transportation of labware, relieving highly trained laboratory staff from logistical duties [81]. With a non-intimidating height (100–160 cm) and organic shapes, Kevin uses a multimodal interface comprising lights, speakers, and a tablet to communicate its status and movement intentions to non-technical personnel. User studies indicate that a “medium” communication level that provides targeted, concise feedback is preferred over continuous signaling to avoid information overload [81].

Clinical specimen delivery systems such as Proxie robot, a mobile collaborative robot (cobot) piloted at Mayo Clinic Laboratories, autonomously move existing carts containing laboratory specimens between pathology stations. This significantly reduces staff effort while maintaining trust through a stable architecture and subtle visual cues, such as expressive “eyes,” which help users feel confident working alongside it. Its “Scout Sense” captures the environment from a human-like eye level to ensure situational awareness, while “Glide 360” mobility allows it to move naturally and intuitively around people. The robot prioritizes safe interaction and uses adaptive AI to learn and harmonize with human workflows, aiming to assist rather than replace staff [82].

Frameworks such as LAPP (Laboratory Automation Plug & Play) further extend flexibility in pharmaceutical and general laboratory automation by enabling mobile manipulator with vision systems to “learn” device poses. This approach aims to simplify the integration of devices from different vendors for end-to-end automation systems [66].

In addition, sophisticated HRI and safety mechanisms are implemented in shared spaces. Mobile systems employ multi-layer smart collision avoidance using Kinect sensors for the real-time recognition of dynamic human face orientation (classified using LVQ neural networks) or specific hand gesture commands (classified using SVM) to receive direct navigational guidance from personnel in narrow zones [65]. Methods to communicate robot intent include projected visual signals, LEDs, speakers, and wearable haptic devices, which improve both predictability and user trust [59].

Research Platforms like the Pioneer 3-DX and TIAGo mobile robots are widely used to study human safety perception and trust [38]. TIAGo, with a semi-humanoid form, an adjustable height, movable head, and multimodal interaction capabilities, supports natural communication via speech, gesture recognition, touch, and emotion detection, allowing safe collaboration in research and healthcare [83].

Overall, indoor mobile robots must balance operational precision, procedural compliance, infrastructure integration, and safe social interaction. Each application, from labware transport to specimen delivery, imposes unique technical and social requirements. Successful deployment depends on integrating perception, socially intelligent navigation, and natural Human–Robot Interaction to meet both safety and user acceptance standards.

#### 3.2.3. Office Environments

Office environments are socially structured but less safety-critical than healthcare or laboratory setting. Robots in offices must navigate within shared workspaces and must respect professional etiquette, meeting norms, and hierarchical structures and team dynamics. Typical tasks include document delivery, providing information services, visitor guidance, and telepresence. The interaction design must minimize disruption and maintain low-friction communication, allowing users to focus on work rather than managing the robot [84].

To assist individuals in office spaces, several research efforts have demonstrated effective HRI solutions. Iida and Abdulali proposed a telepresence robot implementing DEtect, TRAck and FOllow (DETRAFO) algorithm [28] to enable intuitive tracking and following users. Ngo and Nguyen developed a cost-effective on-device Natural Language Command Navigation System for mobile robots in indoor environments like an office, making the Human–Robot Interaction efficient and effective to communicate goals via natural commands [85]. Additionally, Balcı and Poncelet introduced movable robotic furniture in shared office spaces to modulate human–human interaction, to avoid distraction, and make spontaneous interaction more meaningful, thereby improving the overall workflow efficiency [86,87].

#### 3.2.4. Industrial Settings

Industrial indoor environments focus on productivity enhancement, repetitive task execution, and safe human–robot collaboration. Cao and Tam proposed a search and fetch operation for mobile manipulation robots that leverages multimodal Human–Robot Interaction via gesture, voice, and face recognition. This can address the persistent labor shortage in roles that require complex, repetitive tasks in indoor environments such as manufacturing factories [12]. Colceriu and Theis examined human-centric GUI designs for mobile cobots to increase their potential for assembly work in industry settings [88]. Huy and Vietcheslav further developed a novel interface framework for Human–Robot Interaction in industry, employing a laser-writer in combination with a see-through head-mounted display using augmented reality and spatial augmented reality to securely exchange information. They also introduced a novel handheld device enabling multiple input modalities, allowing users to interact with mobile robots’ efficiency [89].

These approaches aim to enhance productivity and safety in harsh or hazardous environments where robots can take over risky jobs [90]. HRI thus plays a central role in improving operational efficiency while maintaining safety in complex indoor construction sites. In such settings, robots work alongside human teams, supporting physically demanding operations, facilitating task communication, and adhering to strict safety protocols [91]. Compared to healthcare or laboratory environments, emotional intelligence and social or companion functions are largely unnecessary. The focus is instead on task performance, reliability, and predictable interaction.

#### 3.2.5. Residential Homes

Residential environments introduce substantial social and behavioral challenges. Robots must navigate shared spaces occupied by multiple individuals with varying routines, preferences, and technical skills [92]. They are required to interpret informal social cues, avoid interfering with family interactions, and respect privacy, including restricted access to certain rooms and protection of personal data. In addition to assistance tasks, residential robots frequently perform companion roles, providing emotional support, engagement through conversation or play, and help with daily routines, particularly for elderly or disabled residents [93].

Monitoring and surveillance tasks are also common in these settings, including autonomous patrolling, security enforcement, and environmental monitoring [94]. While these applications are socially interactive and assistive [57,95,96], they raise critical privacy and ethical considerations, as robots often collect sensitive data about people and spaces. Designers must carefully implement robust data protection, consent mechanisms, and transparent reporting, to ensure that the robot’s presence does not infringe on individual rights or create distrust. Social and companion roles are increasingly relevant in residential care, home environments, and facilities supporting vulnerable populations. In these roles, robots provide emotional support, companionship, and engagement through conversation, play, or assistance with daily routines [93]. These applications demand the most sophisticated HRI capabilities, including emotion recognition, adaptive personalization, and the ability to build long-term relationships with users. Robots must interpret subtle social cues, respond appropriately to changing moods, and foster trust over repeated interactions, creating a sense of presence and companionship that extends beyond functional task performance. Demand for increased convenience, security monitoring, and remote care for relatives at home can be achieved by the Astro robot from Amazon [97]. Examples of residential robots include the Astro platform from Amazon, which supports convenience, security monitoring, and remote care for relatives at home [97]. Other robots, such as ZERITH H1 and Loki (Loki Robotics), are used for housekeeping and toilet cleaning in homes, hotels, and offices [98,99]. Unlike laboratories or industrial robots, residential robots prioritize social acceptance, privacy preservation, adaptability to unstructured environments, and long-term relational interaction. They require sophisticated HRI capabilities, including emotion recognition, adaptive personalization, and the ability to foster trust over repeated interactions, creating a sense of companionship that extends beyond functional task performance.

### 3.3. Synthesis

Although indoor mobile robots share foundational capabilities such as navigation, perception, and infrastructure integration, the nature and complexity of Human–Robot Interaction (HRI) differs substantially across domains. These differences arise from variations in the environmental structure, user expectations, task criticality, and social context.

In industrial settings, HRI is primarily task-oriented and performance-driven. Interaction focuses on clear command input, predictable system responses, and compliance with safety protocols. Multimodal interfaces (e.g., gesture, voice, GUI, augmented reality) are designed to increase efficiency and reduce cognitive load during repetitive or hazardous operations. Social expressiveness and emotional intelligence are largely unnecessary; instead, transparency, reliability, and unambiguous intent communication are central. The human operator remains goal-directed, and interaction serves operational optimization.

Similarly, laboratory environments require highly structured and controlled interaction. Here, HRI must support precision, traceability, and procedural compliance. Communication is typically concise and purpose-specific, avoiding distraction in cognitively demanding research settings. Robots must signal movement intentions clearly to ensure safety in confined spaces, but excessive social signaling can reduce usability. Trust is established through accuracy, predictability, and seamless integration into laboratory workflows rather than through expressive or companion-like behavior. Thus, HRI in laboratories is functional, minimally intrusive, and tightly coupled to workflow reliability.

In healthcare environments, HRI becomes significantly more complex. Robots interact not only with trained professionals but also with patients, elderly individuals, and visitors. Consequently, interaction must combine clinical precision with social sensitivity. Speech recognition, gesture interpretation, contextual awareness, and user modeling are required to personalize assistance and accommodate varying cognitive and physical abilities. Emotional sensitivity, privacy protection, and trust-building are critical. Unlike in laboratories or industry, interaction failures may directly affect well-being or patient confidence. HRI must therefore be adaptive, transparent, and ethically grounded.

Office environments occupy an intermediate position. While less safety-critical, they are socially structured and norm-sensitive. HRI must be low-friction, socially compliant, and minimally disruptive. Robots should respect the proxemics, meeting etiquette, and hierarchical dynamics. Interaction is often informational (e.g., navigation guidance, telepresence, task delivery) and must be seamlessly integrate into daily routines. Compared to healthcare, emotional engagement is less central; compared to laboratories, social appropriateness carries greater weight than strict procedural precision.

The most demanding HRI requirements emerge in residential environments. Homes are socially dynamic, informal, and privacy-sensitive spaces with heterogeneous users, including children, elderly individuals, and guests. Robots must interpret subtle social cues, adapt to changing routines, and avoid interfering with family interactions. In companion or assistive roles, HRI must support emotion recognition, adaptive personalization, and long-term relationship building. Trust formation, consent management, and data transparency are not peripheral concerns but central design constraints. Unlike industrial or laboratory systems, residential robots are evaluated as much on relational quality and perceived presence as on task performance.

Across domains, several cross-cutting HRI dimensions can be identified:

- Intent Communication: Required everywhere, but ranging from purely functional signaling (industry, labs) to socially expressive behavior (homes, healthcare).
- Adaptivity: Minimal in highly structured environments; essential in healthcare and residential settings.
- User Modeling: Optional in industrial contexts; critical in long-term residential or elderly care scenarios.
- Emotional Intelligence: Marginal in productivity-driven domains; central in companion-oriented applications.
- Trust Formation: Performance-based trust dominates in laboratories and industry, while relational and privacy-based trust becomes decisive in homes and healthcare.

In summary, indoor mobile robotics do not present a uniform HRI problem. Instead, each domain shifts the balance between efficiency, safety, social intelligence, emotional responsiveness, and ethical safeguards. Successful HRI design therefore requires domain-specific prioritization layered upon a shared technical foundation (Table 3 and Table 4).

## 4. The Technical Aspect of HRI

**Figure 4:**
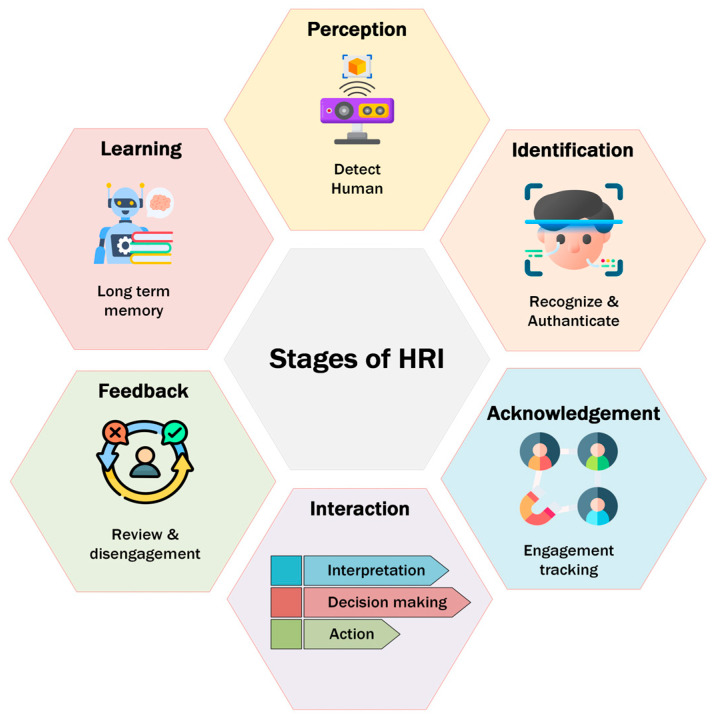
Stages of HRI.

Mobile robots in indoor environments typically perform tasks through three major steps: perception, navigation, and interaction. Among these, the Human–Robot Interaction (HRI) component is central when a robot executes tasks on behalf of or in collaboration with humans. Effective HRI ensures safety, comfort, and efficient task execution, forming the core of human-centered robot behavior (Figure 4).

Before explicit interaction begins, implicit cues during navigation already constitute a form of interaction. For instance, human-aware navigation communicates the robot’s intent, enhancing coordination in shared spaces and providing humans with a sense of safety [34,54,60,100]. Similarly, interaction modalities such as speech, gesture, touch, and visual cues shape how humans understand and guide the robot’s actions.

While many studies have catalogued individual methods and interaction techniques, a structured, analytical synthesis of these approaches is still lacking. Current research often focuses on isolated methods without systematically comparing their robustness, computational requirements, user cognitive load, or adaptability to real-world indoor environments. This gap motivates the present chapter, which aims not only to summarize existing work but also to critically evaluate methods, highlight emerging trends, and identify open challenges and research gaps.

In the following sections, the chapter is organized to support this analytical perspective:

**Human-aware navigation** (Section 4.1)—methods for safe, legible, and socially compliant navigation, including a comparison of classical and learning-based approaches.

**Interaction modalities** (Section 4.2)—detailed treatment of speech, gesture, touch, visual/gaze, and multimodal systems, with structured comparison tables, evaluation of performance trade-offs, and integration of recent advances such as LLMs and VLMs.

**Cross-cutting synthesis** (Section 4.3)—an overview of trends, methodological patterns, and critical limitations across modalities.

By explicitly combining descriptive and analytical perspectives, this chapter aims to move beyond a simple catalog of HRI techniques and provide a critical, structured review of the current state-of-the-art in indoor mobile robotics.

### 4.1. Human-Aware Navigation

Before explicit interaction begins, implicit interaction already occurs through the navigational behavior of the robot. Human-aware navigation aims to give human a perception of safety, communicate the robot’s intent, and facilitate coordination in shared workspaces [34,53,60,100]. Design principles include expressive movement, legibility, comfort, and adherence to social norms in human-populated environments [34]. Table 5 represents four approaches to social navigation.

**Figure 5:**
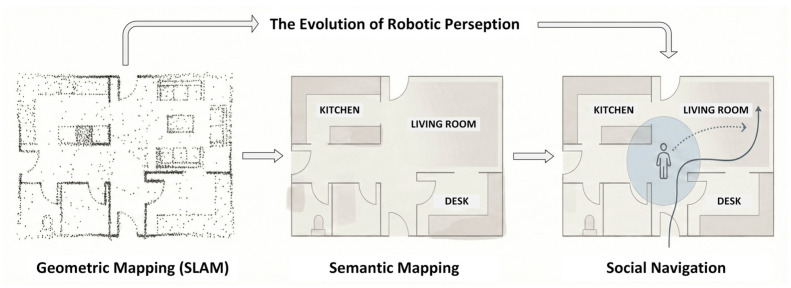
Evolution of robotics navigation.

The human-centric nature of perception and navigation in indoor mobile robots can be seen in Figure 5.

While all four approaches enable human-aware navigation, they differ in robustness, adaptability, computational requirements, and real-world applicability. Reactive approaches are simple and fast but limited in dynamic environments, whereas predictive and model-based approaches improve coordination and social compliance at the cost of higher computational effort. Learning-based methods provide adaptive and socially compliant behaviors but require extensive training and sensor integration. Understanding these trade-offs is crucial for selecting navigation strategies tailored to specific indoor environments and user populations.

**Figure 6:**
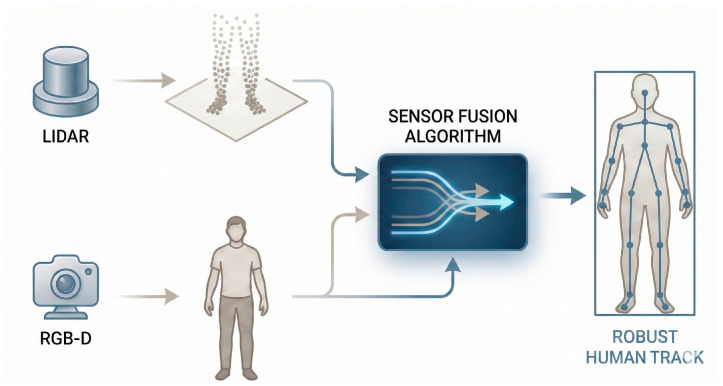
Human tracking and following.

Lasota et al. survey safety strategies for close-proximity collaboration [54], and these principles are integrated into collision avoidance and navigation systems. One of the primary limitations is space; indoor environments often feature narrow corridors, cluttered rooms, and dynamic obstacles, requiring compact, agile robots with highly precise navigation capabilities while maintaining safety and trust of their human counterparts and not becoming a hurdle in their path [55]. Robots must maneuver around furniture, equipment, and humans safely and without disruption. Power management represents another critical challenge [101]. Many robots, especially in healthcare settings or laboratory settings, must operate continuously for extended periods, often 8–12 h, without frequent charging [102]. This necessitates efficient energy consumption, optimized motion planning, and, in some cases, the ability to autonomously dock and recharge. Advanced power systems, lightweight materials, and energy-efficient components are therefore essential design considerations. Human tracking and following is another aspect of assistive robots. Multisensor-based human detection and tracking systems combine data from cameras, depth sensors, and other modalities to achieve robust performance in crowded environments [103]. Intelligent mobility-assistance robots leverage multimodal sensory processing to provide safe and effective support for users with mobility impairments [104]. RGB-D sensor-based real-time people detection and mapping systems enable mobile robots to maintain awareness of human positions and movements [105], helping overcome the limitations of individual modalities and providing redundancy in case of sensor failures. A typical human tracking and following system is presented in Figure 6.

Simultaneous Localization and Mapping (SLAM) is essential for indoor navigation. Robots must accurately map unknown or changing environments while simultaneously tracking their position in real-time. SLAM systems must remain robust in dynamic spaces with moving obstacles, variable lighting, and crowded conditions [73,106]. High-precision mapping supports both task execution and safe interaction with humans. Modern indoor robots employ a variety of sensors, including LiDAR, RGB-D cameras, ultrasonic sensors, and inertial measurement units (IMUs), which must operate reliably across diverse indoor conditions [107]. Collaborative mapping approaches enable multiple robots to work together in constructing environmental representations, improving coverage and efficiency [108]. Advanced scan matching techniques exploiting dominant directional features and improve localization accuracy [109], while integrating Ultra-Wideband (UWB) technology with traditional SLAM systems further enhances indoor positioning accuracy and human avoidance [110].

The evolution from reactive to learning-based navigation reflects a broader trend toward adaptive, socially aware, and context-sensitive navigation. Multisensor fusion, predictive path planning, and collaborative mapping are increasingly standard. However, open challenges remain:

- Robust operation in extremely dense or highly dynamic indoor spaces.
- Trade-offs among computational cost, real-time performance, and social compliance.
- Long-duration operation under constrained energy resources.
- Seamless integration with multimodal interaction modalities to ensure cohesive HRI.
- Adaptability to diverse user populations with varying mobility and cognitive abilities.

Addressing these gaps is critical for the next generation of indoor mobile robots capable of safe, efficient, and socially compliant operation in real-world human environments.

### 4.2. Interaction Modalities

**Figure 7:**
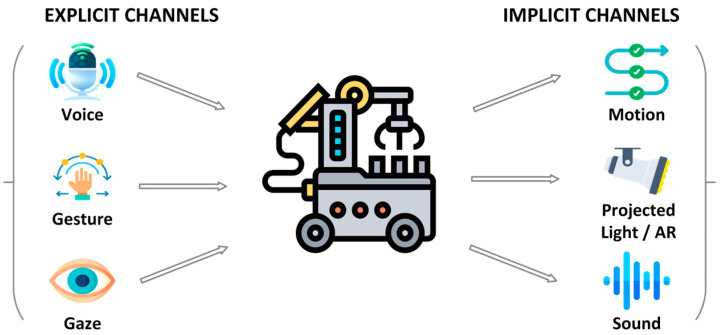
Channels of HRI.

The effectiveness of indoor mobile robots depends critically on the design and implementation of appropriate interaction modalities, as these determine how intuitively, efficiently, and safely humans can communicate with and control robotic systems. Unlike traditional human–computer interfaces, which often rely on static screens or input devices, HRI in mobile robotics must account for the dynamic and content-rich nature of human-centered environments. This includes managing spatial relationships, supporting natural and multimodal communication, adapting to human mobility and movement patterns, and responding to real-time environmental changes that affect both robot behavior and human expectations [111]. Figure 7 depicts the explicit and implicit channels of interaction typically used in indoor mobile robotics. These channels span speech, gesture, touch, visual/gaze, and multimodal approaches, forming the basis for safe, effective, and social-aware interactions.

Domain-specific studies provide valuable insights into user expectations and interaction requirements. Reviews of social robots in classrooms highlight evidence for effective engagement and learning outcomes [61], while research in autism-related therapy demonstrates the potential of robots to support specialized interventions [70]. Similarly, studies of service robots in home environments reveal how everyday adaptations, user preferences, and environmental constraints shape interaction design [4]. These findings emphasize that no single interaction modality suffices across all contexts. Instead, interaction systems must be selected and adapted based on the task, user population, and environmental characteristics. The following Section 4.2.1, Section 4.2.2, Section 4.2.3, Section 4.2.4 and Section 4.2.5 provide a detailed examination of each major modality—speech, gesture, touch, visual/gaze, and multimodal systems—highlighting their technical characteristics, comparative strengths, current trends, and remaining limitations. By systematically evaluating each modality, we aim to provide a critical and structured synthesis rather than a simple catalog of existing methods.

#### 4.2.1. Speech-Based Interaction

Speech-based interaction represents one of the most natural communication modalities for humans and has been extensively studied in indoor mobile robotics [112]. Voice interfaces enable hands-free operation, which is particularly valuable in healthcare and laboratory environments where users’ hands may be occupied with other tasks [113].

**Figure 8:**
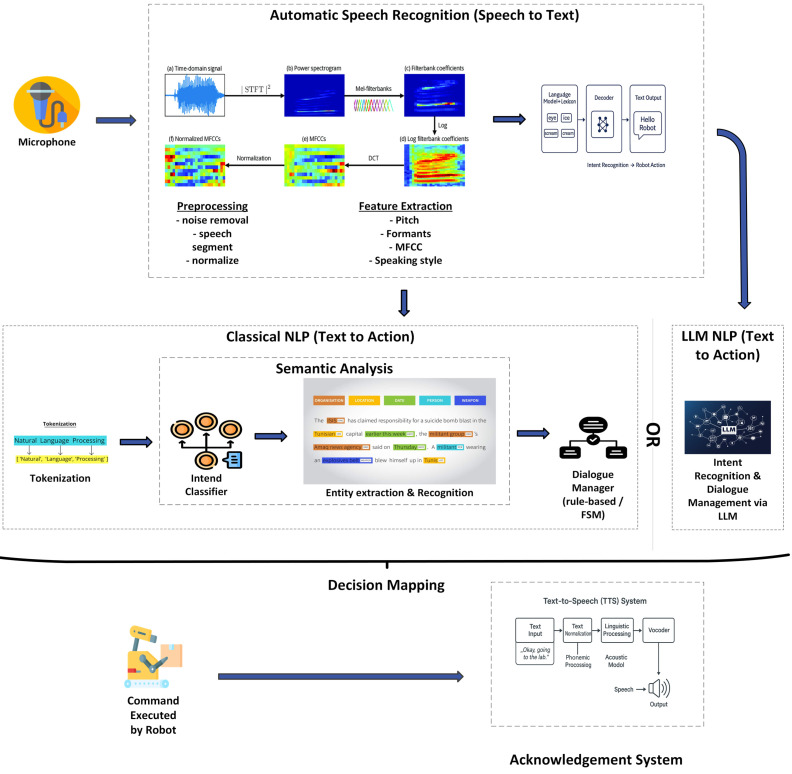
Process of a typical speech-based interaction.

Automatic Speech Recognition (ASR) technology has matured to a level suitable for practical deployment, with modern systems achieving high accuracy even in noisy environments [114]. Cloud-based ASR systems can further improve performance but raise privacy concerns in sensitive settings, leading to increased interest in on-device processing [115]. Figure 8 illustrates the process of a typical speech-based interaction with distinct components like ASR, NLP and TTS.

Natural Language Processing (NLP) allows robots to understand complex commands and engage in contextual conversations [116]. Large Language Models (LLMs) are increasingly integrated into robotic systems, enabling sophisticated dialogue management and intent understanding [117]. Cognitive instruction interfaces leverage natural language understanding to provide intuitive robot navigation commands [118], while recent advances in grounding implicit goal descriptions allow robots to interpret ambiguous spatial references through recursive belief updates [119]. Overall, LLM integration is emerging as a powerful approach to enhance the comprehension of complex verbal instructions [120].

**Figure 9:**
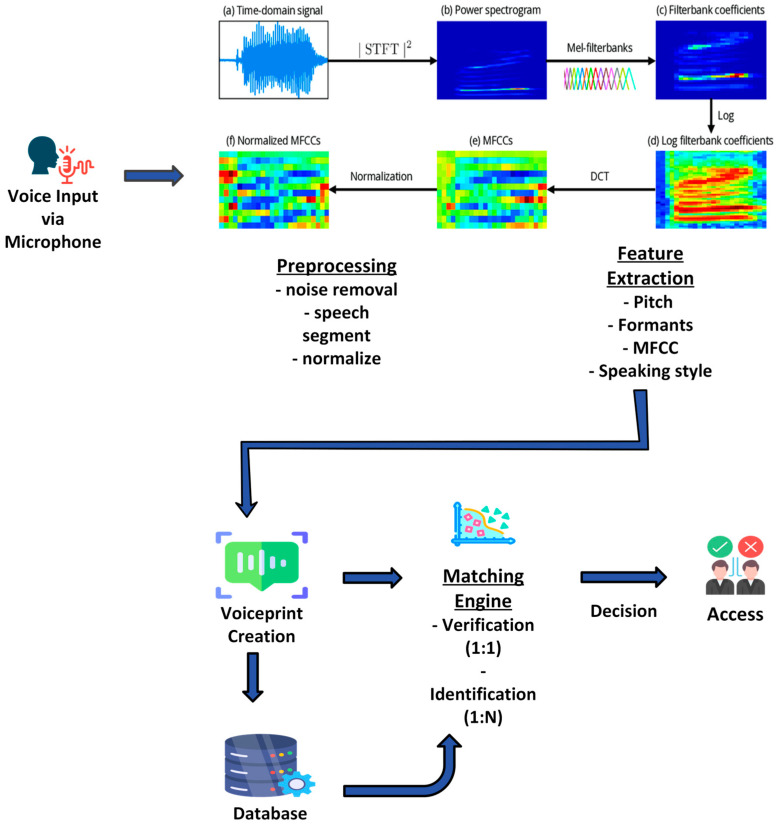
Voice biometric process.

The third component of speech interaction is text-to-speech (TTS). Recent developments in speech synthesis allow robots to provide natural-sounding feedback and convey emotional states [121]. Paralinguistic features such as tone, pace, and volume can communicate robot intentions and emotions, enhancing the overall user experience [122]. For secure interactions, voice biometric authentication ensures that only recognized personnel can control the robot, even when multiple users are present (Figure 9).

Despite these advances, speech-based interaction in indoor environments faces several technical and practical challenges. Ambient noise, multiple speakers, and acoustic reverberation can degrade recognition performance [123]. Cultural and linguistic diversity requires multilingual support and accent adaptation [124]. Privacy concerns are particularly critical when voice data is processed or stored in healthcare settings [125].

Table 6 summarizes the main approaches to speech-based interaction, including classical keyword/grammar-based methods and their key performance characteristics, evaluation environments, metrics, and identified limitations. This structured comparison highlights the trade-offs and gaps in existing methods, motivating the integration of LLM and VLM approaches discussed below.

Recent advances in Large Language Models (LLMs) and vision-language models (VLMs) are fundamentally transforming Human–Robot Interaction. Unlike traditional command-based speech recognition, LLMs enable context-aware reasoning, natural language understanding, and adaptive dialogue, allowing robots to interpret complex user intents and interact in more flexible, human-like ways. Compared to classical interaction modalities such as command-based speech, gesture, or gaze, LLM-driven approaches provide enhanced adaptability, a richer semantic understanding, and the ability to integrate multimodal input streams. This paradigm shift allows indoor mobile robots to operate more autonomously in dynamic environments while improving the naturalness of human–robot collaboration.

Despite these capabilities, deploying LLMs on mobile robots introduces several practical challenges. Onboard inference can be limited by hardware constraints, whereas cloud-based inference introduces network latency and potential reliability concerns. Safety-critical tasks require a careful evaluation of decision timing, error propagation, and fallback mechanisms to ensure robust interaction in real-world environments.

To systematically analyze these methods, we evaluate LLM- and VLM-based approaches using the same criteria applied to classical modalities, including computational cost, robustness to environmental noise, user cognitive load, and hardware requirements. This comparative perspective highlights the trade-offs among performance, deployability, and safety and informs the selection of appropriate speech-based interaction strategies in indoor mobile robotics.

Table 7 summarizes the main LLM- and VLM-based approaches to speech HRI, highlighting their evaluation settings, metrics, strengths, and limitations. Compared to classical methods (Table 6), these approaches offer a richer context understanding, multimodal integration, and greater flexibility, but require the careful consideration of computational cost, latency, and safety-critical constraints.

For a quick comparative overview, Table 8 summarizes key differences across computational costs, robustness, user load, hardware requirements, evaluation metrics, strengths, and limitations, highlighting the evolution from traditional to modern methods.

This comparative overview highlights the trade-offs between classical and modern LLM/VLM-based speech interaction methods, emphasizing where traditional approaches fall short and motivating the integration of context-aware, multimodal models to improve robustness, flexibility, and user experience in indoor mobile HRI.

#### 4.2.2. Gesture-Based Interaction

Gesture-based interaction provides an intuitive and natural communication channel that can complement or substitute for speech in various scenarios [133]. Hand and arm gestures can convey spatial information, directional commands, and social signals that are difficult to express verbally [134].

**Figure 10:**
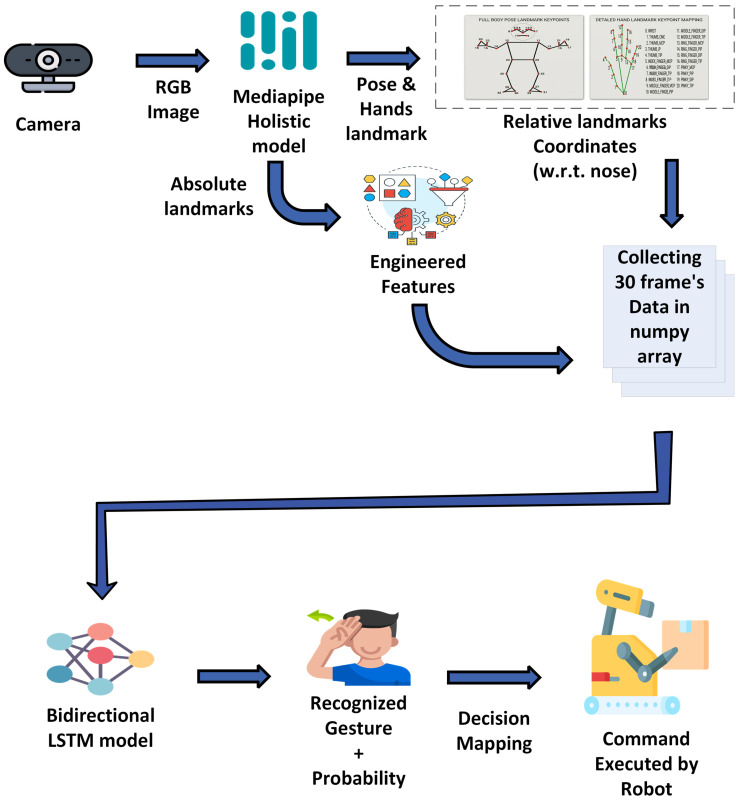
Process of a typical gesture-based interaction.

Vision-based gesture recognition systems primarily rely on RGB cameras, depth sensors, or combinations thereof to capture and interpret human movements [135]. These systems must operate in real-time while remaining robust to variations in lighting, occlusions, user differences, and environmental clutter [136]. A typical gesture-recognition pipeline combines spatial understanding (e.g., MediaPipe Holistic [137]) with temporal modeling via recurrent networks such as LSTMs to interpret dynamic gestures (Figure 10).

Simple gesture vocabularies have proven effective for basic robot control, including pointing gestures for navigation, hand signals for stop/start commands, and waving to attract attention [138]. More complex gestures can convey emotional states, social intentions, and task-specific instructions [139].

Spatial gesture recognition allows users to indicate locations, directions, and objects within the environment [140]. Over time, gesture-based HRI methods have evolved from template- or rule-based systems toward machine learning and deep learning approaches. Machine learning-based systems enable robots to recognize subtle gestural cues and adapt to user-specific behaviors, supporting tasks such as mobility assistance [141]. Deep learning methods, including LSTM or Transformer models, further enhance recognition accuracy and temporal understanding, enabling real-time gesture control in dynamic environments [142]. Collaborative object handling between robots and human operators also benefit from sophisticated gesture recognition and motion prediction [143], which is particularly valuable for mobile robots navigating to specific locations or interacting with environmental objects. Overall, the field shows a clear trend from fixed vocabularies to adaptive, learning-based systems, capable of interpreting complex gestures, social cues, and emotional states. Table 9 provides a summary of gesture-based HRI methods including suitable evaluation metrics, and limitations.

Despite these advances, gesture-based interaction faces several challenges. Gesture ambiguity and cultural differences can lead to misinterpretation [144], and environmental factors, such as lighting, background clutter, and camera positioning, strongly influence recognition reliability. Many systems require user training or adaptation to function robustly. Furthermore, computational cost and latency for real-time deep learning models can constrain deployment on mobile robots. Finally, integrating gesture recognition with other interaction modalities, such as speech or gaze, remains an open area of research to improve robustness, flexibility, and overall user experience.

#### 4.2.3. Touch and Physical Interaction

Touch and physical interaction provide direct, immediate feedback channels that are particularly valuable for users with visual or auditory impairments [145]. In contrast to speech or vision-based modalities, tactile interaction enables unambiguous, proximal control and often reduces the cognitive load, especially in safety-critical scenarios. Tactile interfaces on mobile robots include touchscreens, physical buttons, force-sensitive surfaces, and haptic feedback mechanisms.

Touchscreen interfaces offer familiar interaction paradigms from smartphones and tablets, enabling complex command input and information display [146]. However, robot mobility introduces challenges. Screen visibility can be compromised during movement, accessibility may vary depending on the robot’s height and orientation, and hardware must withstand diverse indoor environments. Thus, while touchscreens support rich interaction, their usability is dependent on context.

The physical manipulation of robot components, such as guiding arm movement or adjusting robot positioning, enables direct spatial instruction [147]. This modality is particularly useful for demonstrating desired behaviors, correcting robot actions, or guiding motion in shared workspaces. Compared to indirect modalities such as speed, physical interaction offers high precision and immediate feedback but requires close proximity and careful safety control.

Haptic feedback through vibration, force, or texture variation can provide the confirmation of user inputs and convey robot status information [145]. Such feedback is especially important for users with visual impairments or in environments where visual attention must be directed elsewhere. In this sense, haptic feedback not only supports accessibility but also enhances situational awareness.

**Figure 11:**
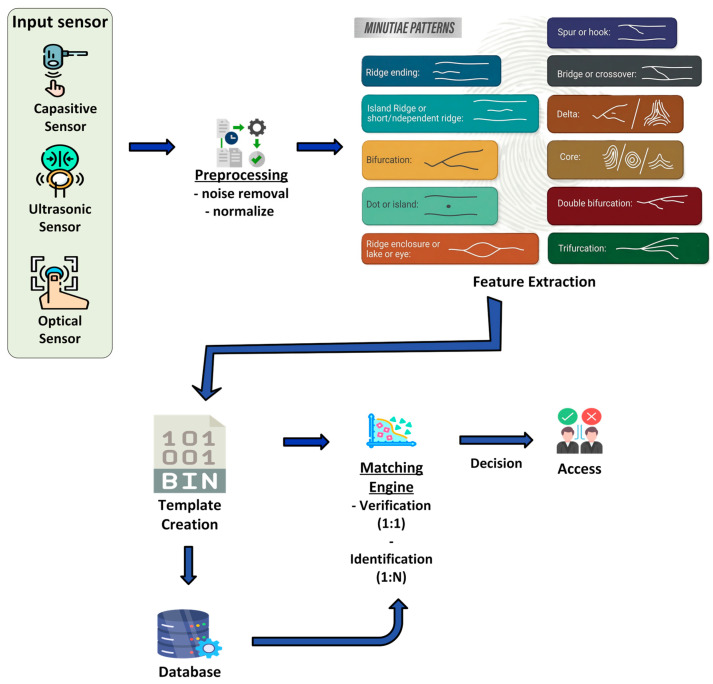
Fingerprint-identification process.

Safety considerations are paramount in the design of physical interactions. Force limitation, emergency stop mechanisms, and collision avoidance strategies must be implemented to prevent injury and ensure safe operation [148]. The robot’s physical design must carefully balance interaction capabilities with user safety requirements. Authentication mechanisms, such as fingerprint identification, ensure that only authorized personnel can access certain touch-based functionalities. Figure 11 represents a typical fingerprint-identification process.

A clear trend can be observed from simple mechanical interfaces toward sensor-rich, force-aware systems that enable compliant and safe physical interaction. Modern systems increasingly integrate force-torque sensors, tactile skins, and adaptive control strategies to support shared control and collaborative behaviors. However, this evolution introduces trade-offs among the hardware complexity, safety certification requirements, and deployment cost.

Despite their intuitive nature, touch and physical interaction modalities face several limitations:

- Requirement of physical proximity, limiting scalability and remote use.
- Safety risks in case of control failure or excessive force.
- Hardware wear and durability issues in high-frequency use scenarios.
- Hygiene and contamination concerns in healthcare environments.
- Limited expressiveness compared to speech or multimodal interaction.

Furthermore, the integration of physical interaction with other modalities (speech, gaze, gesture) remains an important research direction to enable seamless transitions between direct and indirect control paradigms.

Table 10 provides a summary of touch and physical interaction HRI methods including evaluation metrics, and limitations.

#### 4.2.4. Visual and Gaze-Based Interaction

Visual interaction modalities leverage human visual perception and attention mechanisms to establish effective communication channels between humans and robots [149]. Eye gaze patterns, facial expressions, and body language provide rich information about user intentions, emotional states, and situational context. These modalities complement speech and touch, enabling more nuanced and socially aware interaction.

**Figure 12:**
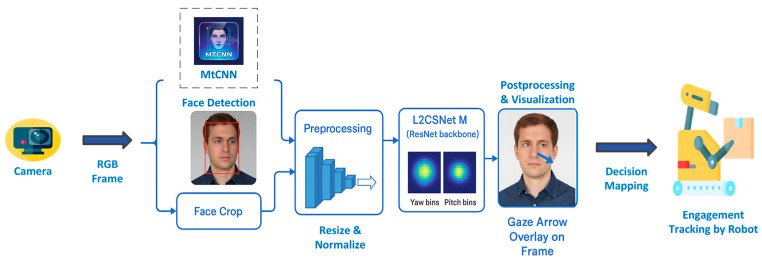
Process of a typical gaze-based interaction.

Gaze-based interaction allows users to direct robot attention, indicate objects of interest, and provide spatial references [150]. Eye-tracking technology integrated into mobile robots can detect the gaze direction and duration, enabling attention-aware behaviors and improving task coordination in shared instances. Figure 12 depicts a typical gaze-based interaction pipeline.

**Figure 13:**
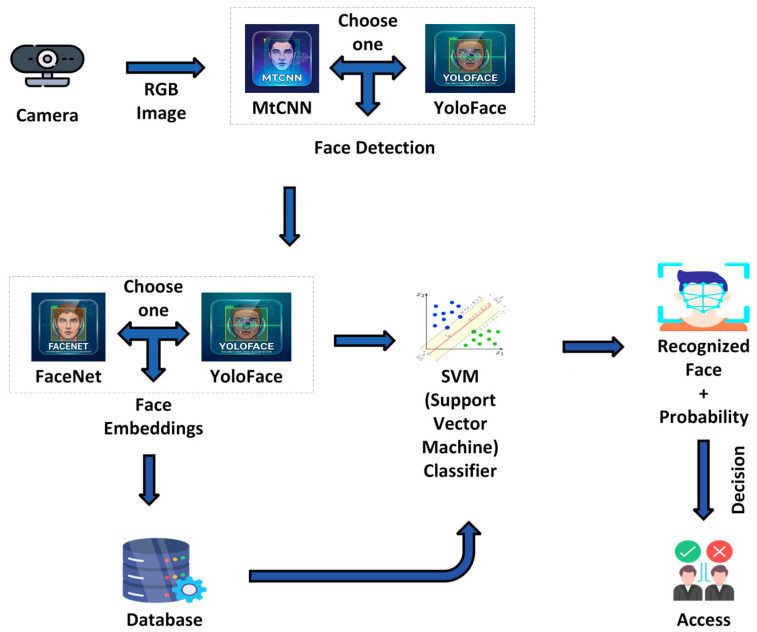
Face biometric process.

Facial expression recognition enables robots to assess user emotional states and adapt the behavior accordingly [151], which is particularly valuable in healthcare, social robotics, and assistive environments. Face recognition additionally provides a secure mechanism for user identification, as illustrated in Figure 13.

Visual displays on robots, including LED patterns, screens, and expressive body language, can convey information, intentions, and emotional states to users [58]. Moreover, robot positioning and orientation communicate social intentions and respect personal space, helping to maintain appropriate distances according to proxemic norms [152]. Effective visual interaction thus integrates perception, expressive signaling, and socially aware navigation.

Despite these advantages, visual and gaze-based modalities face several challenges. Variations in lighting, occlusion, and user mobility can degrade recognition performance. Cultural differences in gestural or gaze meaning require adaptive interpretation. Privacy concerns are also paramount when capturing or processing visual data, particularly facial recognition information.

Visual and gaze-based HRI methods can be grouped into several categories:

- Gaze Tracking Systems—Eye trackers and camera-based systems for attention-aware behavior and spatial referencing [150].
- Facial Expression Recognition—Classifiers (traditional or deep learning-based) to infer user affective states and guide robot responses [151].
- Face Recognition and Biometric Access—Security and user identification using facial data [58].
- Visual Feedback Displays—LEDs, expressive screens, or robot motion to communicate information and robot intent [58,152].

A clear trend emerges toward deep learning-based and multimodal visual interpretation, combining gaze, facial expressions, and robot positioning to support context-aware behavior. Integrating these systems with other modalities (speech, gesture, touch) enhances robustness, adaptivity, and user experience.

Table 11 provides a comprehensive summary of visual and gaze-based HRI methods including their approaches, metrics, and limitations. 

#### 4.2.5. Multimodal and Adaptive Systems

Multimodal interaction systems combine multiple input and output modalities to create more robust and flexible communication channels [153]. These systems can adapt to user preferences, environmental conditions, and task requirements by dynamically selecting appropriate interaction modalities. Multimodal human–robot interfaces that combine speech with other modalities have proven effective for remote robot operation [154]. Sensor fusion techniques combine data from speech, vision, touch, and other sensors to improve recognition accuracy, robustness, and overall system reliability [155].

Adaptive interaction systems learn user preferences and adjust their behavior over time [87]. Machine learning algorithms optimize interaction strategies based on user feedback, task success rates, and environmental conditions. Context-aware systems consider environmental factors, user states, and task requirements when selecting interaction modalities [156]. For example, a robot may switch from speech to visual display in noisy environments or use gesture recognition when users’ hands are free.

The integration of multiple modalities requires sophisticated fusion algorithms and decision-making frameworks [157]. Temporal synchronization, conflict resolution, and priority management are critical considerations in designing robust multimodal HRI systems. Despite advances, challenges remain in the latency, computational cost, sensor calibration, and safety-critical decision making, especially in real-world, dynamic environments.

Table 12 provides a comprehensive comparison of interaction modalities across key performance dimensions, highlighting the complementary nature of different approaches and the importance of multimodal integration for optimal user experience.

Recent advances in vision-language models (VLMs) have enabled multimodal systems to integrate visual perception with natural language understanding, allowing robots to interpret user commands and environmental cues simultaneously. For example, a robot can identify objects in its environment using VLM-based perception such as Contrastive Language-Image Pretraining (CLIP) [132] while understanding instructions provided in natural language through LLM reasoning.

This integration represents a major step toward “embodied AI,” shifting mobile service robots from simple command executors to autonomous, high-level agents capable of intuitive communication and context-aware assistance [158]. By leveraging LLMs and VLMs, mobile robots can perform language-conditioned control, allowing them to navigate via complex semantic instructions (e.g., systems like NavGPT [159]) and execute adaptive mobile manipulation tasks (e.g., BUMBLE) [76]. To ensure continuous and seamless Human–Robot Interaction (HRI), novel frameworks utilizing Perception-Action Loops (PALoop) have been introduced, enabling robots to combine logic reasoning with pre-trained databases for long-horizon planning [160].

A critical challenge in applying these foundation models to physical environments is bridging the gap between abstract text and the 3D world. To address this, frameworks like the TAsk Planing Agent (TaPA) align LLMs with open-vocabulary visual object detectors to generate grounded, executable plans for complex indoor household tasks [161]. Similarly, 3D vision-language-action (VLA) models, such as LEO, allow mobile agents to perceive, reason, and act directly within 3D spaces, responding accurately to multi-round user instructions [162]. Because “hallucination” in LLMs poses a safety risk in physical HRI, recent prompting mechanisms like Contextual Set-of-Mark (ConSoM) have been developed to significantly improve visual grounding and precision in indoor robotic scenarios [163].

Furthermore, modern VLM integration is expanding beyond simple RGB vision to true multisensory perception. Advanced models like MultiPLY integrate 3D visual data with tactile, auditory, and thermal inputs [164]. This multisensory approach is pivotal for robust embodied interaction; for instance, incorporating tactile sensing enables a mobile manipulator to gather vital safety feedback to avoid applying excessive force, while auditory processing allows it to isolate voice commands [165]. Finally, to ensure safety and precision during task execution, researchers are implementing closed-loop feedback systems. By integrating Small Language Models (SLMs) alongside VLMs, indoor mobile robots can iteratively evaluate scene changes and refine their control commands in real-time, adapting instantly to dynamic human environments [166].

Table 13 provides a comparative overview of multimodal and VLM-based interaction methods. Each approach is evaluated across key dimensions including computational cost, robustness to noise, user cognitive load, and hardware requirements. This table highlights the advantages of combining multiple modalities with LLM/VLM reasoning, such as increased adaptability, improved semantic understanding, and enhanced task performance. At the same time, it draws attention to limitations such as latency, onboard resource demands, and safety-critical constraints, guiding design decisions for real-world deployments.

Integrating LLMs and VLMs into multimodal interfaces represents a paradigm shift in HRI, moving from fixed-rule fusion strategies toward adaptive, context-aware reasoning systems. This enables more natural, flexible, and socially compliant human–robot collaboration. Design decisions for selecting modalities or combinations thereof should be guided by the task context, user population, and environmental constraints, with explicit consideration of computational limitations and safety-critical requirements.

### 4.3. Cross-Cutting Synthesis

Human–Robot Interaction in indoor mobile robotics encompasses multiple modalities, each offering distinct advantages, constraints, and suitability depending on the context. While previous sections have discussed speech, gesture, touch, visual/gaze, and multimodal approaches separately, synthesizing these insights highlights overarching trends, comparative strengths, and persistent challenges.

Table 14 provides a comprehensive overview of interaction modalities, highlighting advantages, limitations, computational demands, and typical applications. This allows for a side-by-side comparison of classical and learning-based approaches, including the role of LLM and VLM reasoning in modern adaptive systems.

A key development in HRI is the clear shift from classical, rule-based systems toward learning-based, adaptive approaches. Early systems for speech and gesture often relied on predefined templates and command sets. Modern deep learning architectures—particularly LSTM and Transformer models—allow the recognition of complex, context-dependent commands. When combined with LLMs and VLMs, robots can now interpret multimodal inputs and respond to subtle, situational user intentions.

Another significant trend is context- and environment-aware adaptation. Multimodal systems integrating speech, gestures, visual, and tactile signals can dynamically select the most appropriate interaction modality. For instance, robots may rely on visual displays or gestures in noisy environments or leverage gesture recognition when users’ hands are free, ensuring both efficiency and clarity of interaction.

Human-centered design is increasingly emphasized. Effective systems feature legible motion, socially compliant behavior, and adaptive responses. Multimodal integration not only enhances efficiency but also accessibility: limitations of individual modalities—such as reduced gesture recognizability in poor lighting or speech commands in visually cluttered environments—can be mitigated through sensor fusion.

Finally, cross-cutting challenges span the entire field. These include dependence on environmental conditions, variability of human users (e.g., cognitive load, cultural differences), and high computational and hardware demands for LLM/VLM-based systems. Moreover, the lack of standardized datasets, evaluation metrics, and protocols complicates the comparative assessment and reproducibility of approaches.

In summary, Human–Robot Interaction in indoor mobile robotics is shaped by evolving methodologies, context-specific applications, and enduring challenges. Understanding these cross-cutting factors is critical for developing adaptive, robust, and user-centered systems and provides a foundation for advancing future HRI research and deployment.

## 5. The Human Aspect in HRI

### 5.1. User Experience and Acceptance

**Figure 14:**
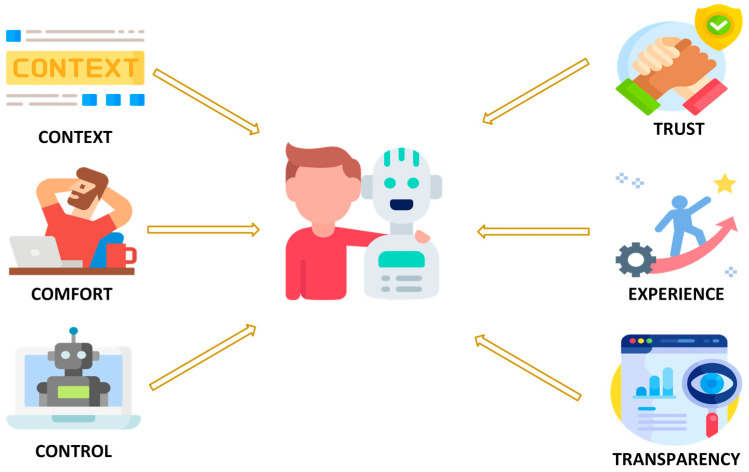
Various human factors affecting robot acceptance.

User experience and acceptance are critical determinants of successful indoor mobile robot deployment, as they shape not only the immediate usability of robotic systems but also their long-term integration into everyday human environments [167]. Beyond technical performance, these factors encompass usability, trust, social acceptance, and the development of a long-term human–robot relationship, all of which influence whether users feel comfortable, empowered, and willing to cooperate with robots. Figure 14 represents various human factors that affect robot acceptance.

Moreover, sustainable adoption also requires attention to relationship building, cultural sensitivity, and the adaptation of robotic behaviors to diverse user groups and contexts, ensuring that technology serves human needs rather than imposing additional burdens. Fong et al. review socially interactive robots and taxonomies of embodiment and modalities [57]. Dautenhahn highlights HRI dimensions and the need for “robotiquette” [95], which is a set of social rules and behaviors that are comfortable, predictable, and acceptable to humans when the robots become social partners, while Feil-Seifer and Matari’c define socially assistive robotics that support users through social (non-contact) interaction [96]. This social perspective complements the technical interaction design, emphasizing the importance of robot embodiment and adherence to social norms in human–robot collaboration.

#### 5.1.1. Human-Centered Design Principles

**Figure 15:**
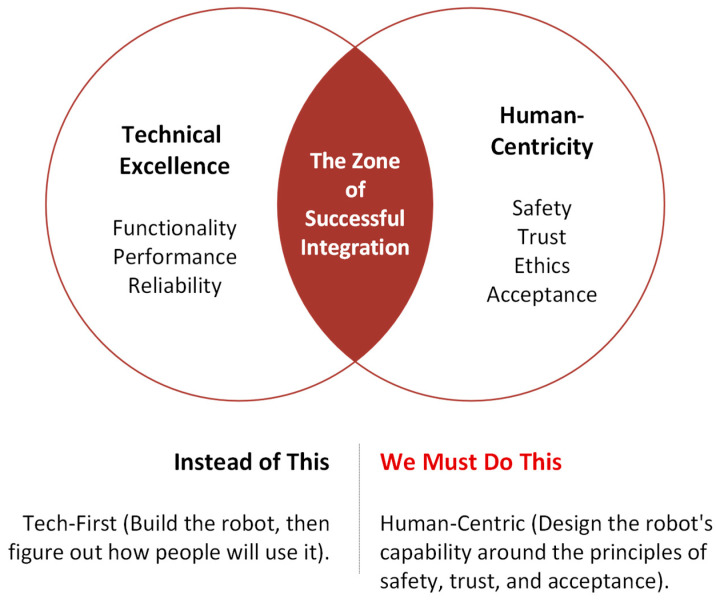
Human-centric design for successful integration.

Human-centered design in indoor mobile robotics requires an understanding of user needs, capabilities, and limitations across diverse populations [168]. This approach emphasizes iterative design, the integration of user feedback, and accessibility considerations throughout the development process. Universal design principles promote robot accessibility for users with varying abilities, ages, and technical expertise [169], including accommodations for visual, auditory, motor, and cognitive impairments. The Dual nature of HRI is depicted in Figure 15.

User interface design must balance simplicity with functionality, providing intuitive controls while enabling complex task execution [170]. The challenge is to accommodate both novice and expert users within a single interaction framework.

Personalization allows robots to adapt to individual user preferences, communication styles, and task patterns [171], achieved either through explicit configuration or implicit learning from interaction behaviors.

#### 5.1.2. Trust and Reliability

Trust is a fundamental requirement for effective Human–Robot Interaction, particularly in indoor environments where robots operate close to humans. Trust calibration and appropriate reliance are central to long-term deployment [36]. Sheridan outlines challenges related to supervision, safety, and value alignment [37]. A meta-analysis quantifies robot performance and attributes as dominant predictors of trust in HRI [35,77]. According to this meta-analysis by Hancock et al., the foundational framework for trust is affected by three major factors in decreasing order of significance:

- Robot-related factors: performance (reliability, capability, and predictability) and attributes (appearance and personality)
- Environment-related factors: task type, team composition and risk level
- Human-related factors: personality, culture, experience, age and gender

**Figure 16:**
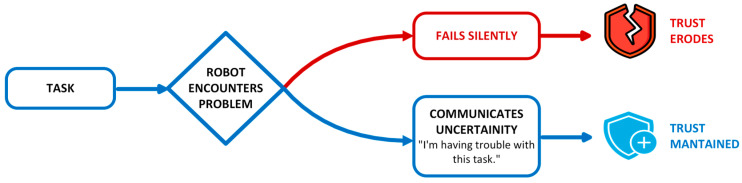
Impact of failure on trust.

System reliability encompasses both technical performance and interaction consistency [172,173]. Users must trust that robots will execute tasks correctly, respond appropriately to commands, and operate safely. Failures or unpredictable behaviors can severely undermine user trust. Much like human relationships, trust in robots is positively affected by their perceived competence (ability to perform tasks effectively) and warmth (compatible intentions, social behavior) [174]. Figure 16 depicts the importance of communication when encountering failure to mitigate or exacerbate the impact. Robots that use explicit and implicit communication to make their actions understandable and predictable are perceived as more trustworthy [59].

Transparency and explainability enable users to understand robot decision-making processes and limitations [36]. This understanding is particularly important when robots make autonomous decisions that affect user safety or task outcomes.

Appropriate trust calibration prevents both over-reliance and under-utilization of robotic systems [124]. Users should maintain realistic expectations of robot capabilities while feeling confident in the robot’s capabilities. Table 15 depicts the translation of human–human trust factors to human–robot trust paradigms.

Cultural factors influence trust development, technology acceptance, authority relationships, and social interaction norms vary across different populations [124]. Robot design must account for these cultural differences to ensure successful deployment in diverse environments.

**Figure 17:**
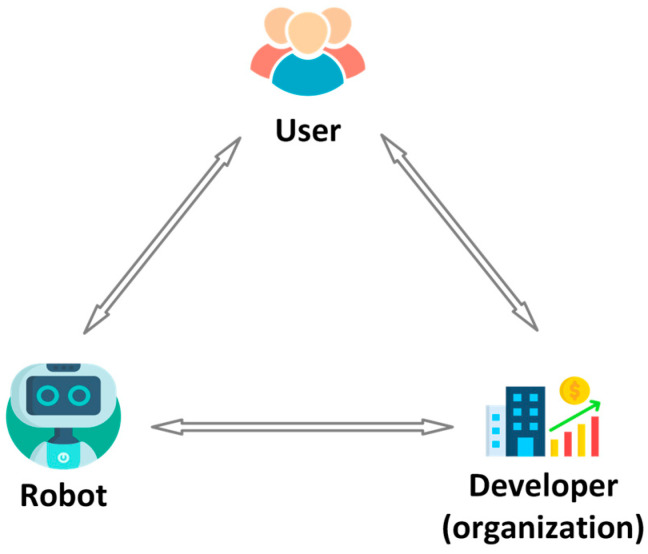
The social triad.

Trust is not a simple dyadic relationship. It is a triad among the user, the robot, and the developer (the organization). A user’s trust in the robot is contingent on their trust in the deploying organization [175]. Figure 17 represents this three-way trust equation.

#### 5.1.3. Social Acceptance and Integration

Social acceptance reflects the broader community’s willingness to integrate robots into shared spaces and daily activities [176]. The Technology Acceptance Model (TAM) suggests that acceptance is driven by perceived usefulness and perceived ease of use [177]. This acceptance depends on perceived benefits, social norms, and ethical considerations. For specific demographics like older adults, psychosocial factors and pre-existing attitudes towards technology play a significant role [63].

Robot appearance and behavior strongly influence social acceptance, with design choices affecting perceived intelligence, friendliness, and trustworthiness [178]. The “uncanny valley” phenomenon suggests that robots should appear either clearly non-human or convincingly human-like to maximize acceptance [179].

Defining the social role of robots establishes appropriate expectations and boundaries for behavior [58]. Clear role definitions prevent role confusion and establish consistent interaction protocols.

Long-term effects must also be considered, as users develop relationships with robots over time [180]. The initial novelty may fade, requiring sustained engagement strategies and the continued demonstration of value.

Successful community integration involves training, support, and change management processes to facilitate robot adoption [14]. Adaptation depends on buy-in from multiple stakeholders, including the users, administrators, and support staff.

Human–Robot Interaction (HRI) studies indicate that user acceptance relies not only on functional capabilities but also on social and emotional factors. Robots that display appropriate social behaviors, maintain culturally sensitive interaction patterns, and adapt to individual user preferences achieve higher levels of acceptance and sustained engagement. The cognitive aspects of HRI extend beyond simple command-response paradigms to include learning, adaptation, and the development of shared mental models between humans and robots [181].

#### 5.1.4. Usability and Accessibility

Usability evaluations in mobile robotics require specialized metrics that account for spatial interaction, mobility, and multi-user scenarios [40]. Traditional usability measures must be adapted for the unique characteristics of mobile robot interaction. The usability heavily depends on the cognitive workload of the supervisor or user. To scale multi-robot systems, the focus must shift from adding more robots to designing systems that reduce the number of commands required per robot and optimize the human interaction model to manage attention and workload effectively [62]. This can be achieved by delegating most of the tasks to a fleet management system, and supervision is done by the user instead of direct instructional control, just like in the case of human management.

Accessibility considerations ensure that robots can effectively interact with users with diverse abilities and needs [6], including support for visual, auditory, motor, and cognitive impairments.

Learnability and memorability are critical, as users must quickly understand robot capabilities and remember interaction procedures [168]. Complex interaction sequences should be minimized, and consistent paradigms should be maintained across different robot functions.

Error prevention and recovery mechanisms help users avoid mistakes and recover from interaction failures [182]. Clear feedback, confirmation dialogs, and undo capabilities can improve user confidence and task success rates.

Performance metrics should include both objective measures (e.g., task completion time, error rates) and subjective measures (e.g., satisfaction, comfort, perceived usefulness) to provide a comprehensive usability assessment [167].

#### 5.1.5. Metrics and Evaluation Standards

Steinfeld et al. propose standardized metrics for task-oriented HRI [40]. For social perception, the “Godspeed Questionnaire Series” measures anthropomorphism, animacy, likeability, perceived intelligence, and safety [41]. Such standardized metrics are essential for comparing robot systems across different applications and ensuring the consistent evaluation of user experience.

These considerations collectively support design principles emphasizing calibrated trust and the use of standardized measurement methods to evaluate robots across diverse settings and applications.

### 5.2. Safety and Privacy

Safety and privacy considerations are paramount in indoor mobile robotics, where robots operate close to humans, navigate complex and dynamic shared spaces, and may access, process, or transmit sensitive personal or institutional information [54]. These considerations encompass not only physical safety—such as collision avoidance, reliable obstacle detection, and fail-safe emergency mechanisms—but also data privacy, including responsible handling of user information, secure communication channels, and transparent data practices. Regulatory compliance also plays an essential role, as robots must adhere to established standards, ethical guidelines, and legal frameworks that protect individuals and ensure accountability for developers and operators. Together, these aspects form the foundation for user trust and the sustainable deployment of mobile robots in human-centered environments.

#### 5.2.1. Physical Safety vs. Perceived Safety

The concept of physical safety in indoor mobile robotics involves preventing harm to humans through collision avoidance, force limitation, and emergency response capabilities and understanding injury mechanisms through systematic collision tests [148], while perceived safety is the perception of humans towards the robot, and it depends on human factors. Safety requirements vary depending on the environment, user population, and robot capabilities.

**Physical Safety:** Multi-sensor approaches combining LiDAR, cameras, and ultrasonic sensors provide redundancy and improve detection reliability. Collision-avoidance systems must reliably operate in dynamic environments with moving humans, furniture, and other obstacles [34]. Human-aware navigation algorithms consider social conventions, personal space, and movement prediction to enable safe and socially appropriate robot behavior [52]. These algorithms must balance efficiency with social acceptability.

Emergency stop capabilities and fail-safe mechanisms ensure that robots can be quickly disabled in case of malfunction or unsafe situations [183]. These systems must be easily accessible and intuitive for users to operate under stress.

Force limitation and compliance control prevent injury during physical interactions, which is particularly important for robots with manipulation capabilities [148]. Robots must be designed to limit forces to safe levels while maintaining sufficient capability for useful tasks.

The identification of dynamic contact-avoidance zones in human–robot collaborative workspaces is crucial for preventing collisions and ensuring safe interaction [184]. Longterm deployments such as the Xavier robot project have provided valuable lessons on maintaining safe operation over extended periods [185]. The detection and state estimation of moving objects from a moving platform remain challenging problems for safe indoor navigation [56].

**Perceived safety**, which may differ from objective safety metrics, significantly influences user acceptance and must be carefully considered in robot design [186]. A robot that is physically safe by design can still be perceived as unsafe by humans. Akalin et al. addressed these influencing factors, which are as follows [187]:

- Comfort: How at ease does the person feel?
- Familiarity: Prior experience with robots.
- Sense of Control: The user’s feeling of agency over the interaction.
- Transparency and Predictability: Clear understanding of the robot’s actions and intentions.

#### 5.2.2. Regulatory and Standards Framework

**Figure 18:**
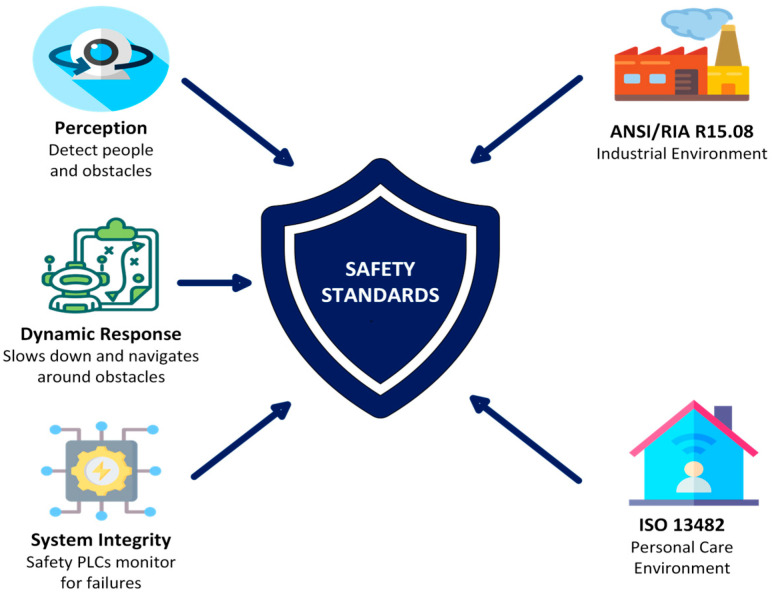
Safety standards.

Safety standards for mobile robots are evolving to address the unique challenges of indoor Human–Robot Interaction. Standards such as ISO 13482 for personal care robots provide frameworks for safety assessments [188]. Physical safety is governed by standards like ISO 15066, which provide guidelines for collaborative robot systems to mitigate hazards from unintended contact [189]. Figure 18 depicts various safety standards involving different environments, perceived and physical safety, and social navigation.

Risk-assessment methodologies must consider both systematic and random failures, human factors, and environmental variations [190]. Comprehensive risk analysis should address all phases of robot operation, including deployment, maintenance, and end-of-life.

Certification processes vary by region and application domain, with medical device regulations applying to healthcare robots and consumer product standards for residential applications [191]. Manufacturers must navigate complex regulatory landscapes to achieve market approval [68].

Liability frameworks remain unclear in many jurisdictions, raising questions about responsibility for robot actions and decisions [192]. Clear legal frameworks are needed to support widespread robot deployment while protecting users and manufacturers.

#### 5.2.3. Privacy and Data Protection

Privacy protection in indoor mobile robots involves safeguarding personal information collected through sensors, interactions, and environmental monitoring [94]. Privacy considerations encompass data collection, storage, processing, access, and sharing practices.

**Figure 19:**
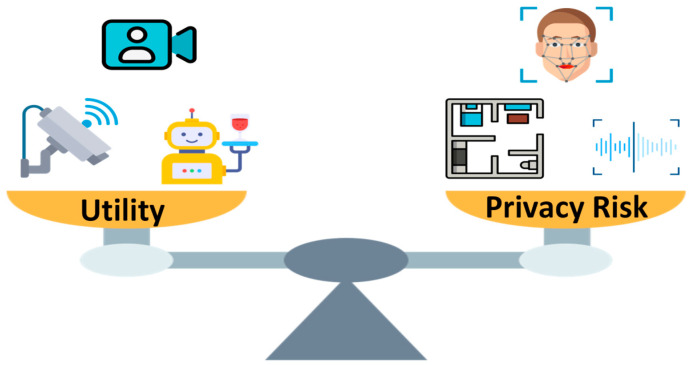
The data dilemma.

Data-minimization principles suggest collecting only necessary information for robot operation and interaction [193]. This approach, which is called privacy by design, reduces privacy risks while maintaining functional capabilities. This privacy versus utility tradeoff is depicted in Figure 19.

Consent mechanisms must be designed to inform users about data collection practices and provide meaningful choices about privacy settings [194]. Dynamic consent systems may be needed to address changing privacy preferences and robot capabilities.

Anonymization and encryption techniques can protect sensitive data while enabling necessary robot functions [67]. However, the effectiveness of anonymization decreases as datasets become richer and more comprehensive. Which increases the concern for household robots becoming targets of malicious attacks, turning a helpful assistant into a surveillance device.

Edge computing and on-device processing can reduce privacy risks by minimizing data transmission and cloud storage [101]. Privacy considerations are particularly important in person-following applications, where robots must track individuals while respecting their privacy boundaries [195].

Security robots deployed in homes and commercial spaces must balance surveillance capabilities with data-protection requirements [196,197]. This approach may require tradeoffs in processing capability and system performance.

#### 5.2.4. Ethical Considerations

Ethical considerations in indoor mobile robotics encompass autonomy, dignity, transparency, and fairness [198]. These considerations become particularly important in vulnerable populations such as elderly users or patients.

Autonomy preservation ensures that users maintain control over robot behavior and decision-making [10]. Robots should enhance rather than replace human agency and decision-making capabilities.

Dignity preservation requires a respectful interaction design that maintains user privacy, choice, and social status [69]. Robots should not infantilize users or create dependency relationships that undermine human dignity.

Transparency in robot capabilities, limitations, and decision-making processes enables informed user consent and appropriate trust calibration [199]. Users should understand how robots operate and make decisions that affect them.

Fairness and non-discrimination require robot systems to provide equitable service across diverse user populations [198]. Bias in the recognition systems, interaction design, or service delivery must be identified and addressed.

**Figure 20:**
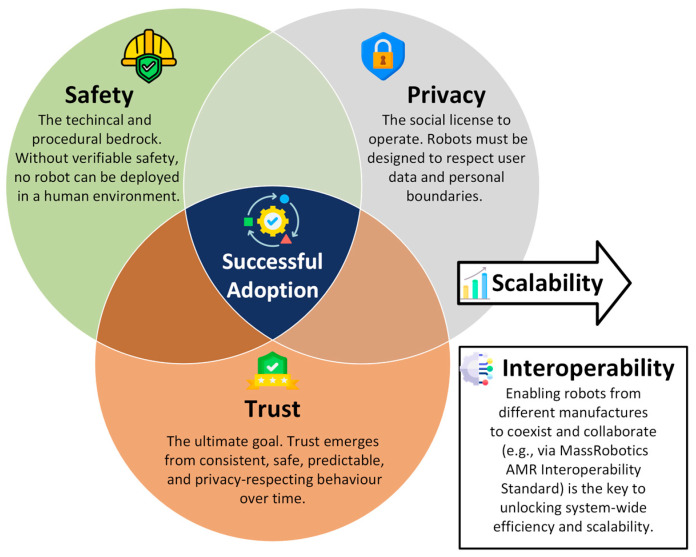
Successful adaptation.

Following landmark HRI research, safety and privacy measures should work together to establish standardized approaches that enable safe, trustworthy human–robot collaboration. Design frameworks should integrate physical safety mechanisms with data-protection protocols and ethical guidelines, supporting consistent assessments across different indoor mobile robot deployments. The success of indoor robotics using Human–Robot Interaction is at the interconnection of these domains, as depicted in Figure 20.

## 6. Case Studies

This section examines three representative indoor mobile robots, Moxi (healthcare), Temi (personal assistant), and Astro (home security/assistance)—to illustrate different approaches to Human–Robot Interaction (HRI) design and implementation in real-world applications. Each system demonstrates distinct strategies for integrating navigation, perception, interaction modalities, and social behaviors, reflecting the specific requirements of their target environments and user populations. By analyzing their interaction design, technical implementation, and user experience, this section highlights the successes and challenges relevant to the development of future indoor mobile robots.

### 6.1. Moxi—Healthcare Assistant Robot from Diligent Robotics

Moxi, developed by Diligent Robotics, represents a sophisticated approach to healthcare robotics with an emphasis on social intelligence and human-aware behavior [71]. Deployed in over 30 hospitals, Moxi has completed more than 500,000 deliveries, demonstrating practical viability in demanding healthcare environments [200].

- Interaction Design: Moxi employs a multimodal interaction approach combining speech recognition, gesture detection, and expressive LED displays [201]. The robot’s anthropomorphic design with animated eyes and head movements creates social presence while maintaining professional appropriateness for healthcare settings.
- Multimodal Efficiency: Hospital environments are notoriously noisy, which can render standard speech recognition unreliable. Moxi compensates for this by utilizing a highly effective multimodal interface (Visual/Gaze + Gestures + Audio cues). Instead of relying solely on voice, Moxi uses animated LED eyes to signal intent, accompanied by auditory “meeps” and specific voice lines to capture staff attention when an intervention is needed and head movements to communicate its status—a direct application of the “Visual/Gaze” advantages outlined in Table 12, providing non-intrusive attention awareness that does not disrupt clinical workflows.
- Technical Implementation: The system integrates advanced navigation capabilities with social awareness, enabling operation in crowded hospital corridors while respecting patient privacy and clinical workflows [202]. Moxi utilizes elevator navigation, badge-access doors, and integration with hospital information systems.
- User Experience: Healthcare staff report positive acceptance due to Moxi’s ability to handle routine tasks (supply delivery, equipment transport) that allow nurses to focus on patient care [2]. The robot’s social behaviors, including greeting staff and expressing gratitude, contribute to positive user relationships.
- Predictability and Transparency: Moxi strongly addresses predictability by strictly adhering to routine hospital corridors and pausing when pathways are blocked to wait for the area to clear. Furthermore, it achieves transparency by using its gaze cues to look in the direction it is about to travel, allowing busy healthcare staff to intuitively anticipate its path and avoid collisions. It exhibits a highly structured transparency loop when facing navigation errors (e.g., failed badge access or network dropouts). Rather than failing silently, it displays explicit error messages on its screen, emits audio alerts, and sends direct status updates to staff communication devices. Furthermore, Moxi utilizes an off-site developer loop using LTE backup networks for remote monitoring. This ensures that while the robot operates autonomously, system failures are highly transparent to both on-site users (for immediate un-blocking) and off-site developers (for lifelong AI training).
- Autonomy and Flexibility: Moxi scores exceptionally high here due to its mobile manipulation capabilities and integration with hospital infrastructure (e.g., calling elevators, opening badge-access doors) [202]. Its ability to rely on emergency stop buttons for manual human intervention ensures flexibility in urgent clinical scenarios.
- Emotional Engagement: Moxi features social behaviors like expressing gratitude or displaying heart-shaped eyes. While this foster perceived warmth and helps staff accept the robot, a persistent challenge remains in maintaining transparency when the robot makes a navigation error, as staff often lack an explainable reason for the failure.
- Challenges and Lessons: Key challenges include integration with existing hospital systems, maintaining sterility protocols, and adapting to diverse clinical environments. In a high-stakes hospital environment, Moxi must be impeccably reliable and safe. Success depends on earning the trust of busy clinical staff and seamlessly integrating into complex, time-sensitive hospital workflows without causing disruption. The success of Moxi demonstrates the importance of task-appropriate design and extensive real-world testing [203].

Healthcare robots must navigate complex regulatory environments while addressing diverse stakeholder needs, including patients, medical staff, administrators, and family members. The integration of mobile robots into clinical workflows requires careful attention to infection control protocols, emergency-response procedures, and interoperability with existing hospital information systems. Brain–computer interface (BCI) technologies are emerging as potential control modalities for patients with severe mobility impairments, enabling direct neural control of assistive robots [20].

### 6.2. Temi—Personal Assistant Robot from Temi

Temi represents a consumer-oriented approach to indoor mobile robotics, focusing on personal assistance, entertainment, and communication [204]. Designed for home and office environments, Temi emphasizes accessibility and ease of use for diverse user populations.

- Interaction Design: Temi features voice-first interaction with natural language processing, complemented by a touchscreen interface and mobile app control [205]. The robot follows users when requested and provides hands-free access to information, communication, and entertainment services [206].
- Multimodal Efficiency: Temi relies heavily on speech-first modality backed by a touch interface (tablet screen). As noted in Table 12, speech provides highly accessible, hands-free interaction, making Temi particularly suitable for elderly users or those with limited mobility [206]. Temi employs an advanced hardware–software audio suite. It utilizes a four-microphone omnidirectional array paired with AI-driven Automatic Speech Recognition (ASR) and Natural Language Processing (NLP). This allows the robot to perform active voice localization—calculating the heading and distance to a specific speaker—while utilizing echo cancellation and noise reduction to filter out background interference. Furthermore, Temi fuses this audio data with facial recognition to visually distinguish between different users, enabling it to isolate commands and provide tailored interactions even in crowded, multi-speaker environments [206]. It relies on cloud-processing latency. The touch screen provides a vital fallback (direct feedback) but requires physical proximity, limiting the robot’s remote utility.
- Technical Implementation: The system utilizes SLAM navigation, face recognition for personalization, and cloud-based AI services for natural language understanding [207]. Integration with smart home systems and video calling capabilities extends the robot’s utility.
- User Experience: Temi targets non-technical users with simple setup and intuitive voice commands [204]. The robot’s friendly appearance and conversational interface reduce technology barriers for elderly users and families.
- Emotional Engagement: Temi succeeds in projecting warmth and approachability through natural language conversations and a non-threatening physical footprint, significantly lowering technology barriers for families. It enhances engagement through physical adaptability—such as the screen automatically adjusting its angle to face the user during telepresence meetings—and broadens its accessibility via multi-language support.
- Autonomy and Flexibility: Unlike Moxi, Temi lacks physical manipulation capabilities. This restricts its flexibility, meaning users must adapt to their expectations for physical assistance. However, its navigational autonomy is highly optimized for dynamic social spaces. Rather than just a “fetch-and-follow” dynamic, Temi’s robust sensor suite allows it to fluidly move between different groups of people in crowded environments (e.g., acting as a guide or sales assistant). Its 20-watt adaptive audio system (incorporating tweeters, mid-range speakers, and a subwoofer) ensures that its communication flexibility remains high even in public settings [207].
- Transparency: Despite its sophisticated localization hardware, a major critical gap for Temi remains the lack of system explainability regarding its always-listening voice activation [205]. Users must implicitly trust the system’s cloud-based processing of their ambient audio and facial data, which can negatively impact long-term dependability and user privacy in intimate domestic spaces.
- Challenges and Lessons: Privacy concerns arise from always-listening voice activation and cloud-based processing [205]. Limited manipulation capabilities restrict task range, highlighting the trade-offs between simplicity and functionality in consumer robotics.

### 6.3. Amazon Astro—Home Security and Assistance

Amazon Astro represents a comprehensive home robotics platform combining security monitoring, family communication, and smart home integration [97]. The system leverages Amazon’s ecosystem of services and devices for seamless integration.

- Interaction Design: Astro employs multimodal interaction, including voice (Alexa), visual displays, and mobile app control [208]. The robot’s expressive screen and movement patterns communicate the status and intentions to family members.
- Multimodal Efficiency: Astro is a prime example of a computationally intensive multimodal system. It fuses voice commands, visual screen expressions, and ambient sensing [208]. By integrating Ring camera technology, Astro extends its visual modality beyond simple HRI into active environmental monitoring [209]. However, as indicated in Table 12, this level of comprehensive sensing in a domestic setting severely exacerbates privacy concerns.
- Technical Implementation: Advanced SLAM technology enables the autonomous navigation and mapping of home environments [209]. Integration with Ring security systems, smart home devices, and Amazon services provides comprehensive home automation.
- User Experience: Astro aims to provide peace of mind through security monitoring while maintaining family connectivity [210]. The robot’s ability to patrol homes, check on elderly family members, and provide video calling enhances its value proposition.
- Predictability: Astro scores well on predictability regarding its autonomous patrols and scheduled routines, leveraging advanced SLAM to reliably navigate changing home environments.
- Emotional Engagement: Astro attempts to build emotional engagement using on-screen digital eyes to mimic animal-like companion behaviors. While Astro succeeds in providing immediate hardware transparency, long-term trust remains a challenge due to its integration with the broader Amazon ecosystem. Although users can manage data via Alexa Privacy Settings, the inherent nature of cloud-based visual and audio processing in intimate domestic spaces creates friction regarding data sincerity and privacy [211], highlighting the ongoing tension between advanced capability and consumer privacy.
- Transparency: A major challenge in domestic robots is the user’s fear of covert surveillance. Astro explicitly addresses this Transparency requirement through distinct hardware cues: a bright green light on its periscope indicates active video streaming, while a blue light and on-screen “picture-in-picture” visuals indicate audio processing. Additionally, physical privacy controls (camera/mic off buttons) and distinct app states (Away/Home/Disarmed) allow users to accurately predict and control the robot’s monitoring behavior.
- Challenges and Lessons: Privacy concerns are significant given Amazon’s data-collection practices and always-on monitoring capabilities [211]. The high cost and limited availability restrict market adoption, illustrating challenges in consumer robot economics.

### 6.4. Comparative Analysis

These case studies illustrate different approaches to indoor mobile robot design, each optimized for specific environments and user needs (Table 16). Moxi’s success in healthcare demonstrates the value of domain-specific design and extensive field-testing. Temi’s consumer focus highlights the importance of simplicity and accessibility in personal robotics. Astro’s comprehensive platform approach shows the potential for ecosystem integration while raising significant privacy concerns. Common success factors include reliable navigation, appropriate social behaviors, and clear value propositions for specific use cases. All three systems face challenges related to privacy, costs, and user acceptance that must be addressed for broader adoption.

## 7. Current Challenges and Future Perspectives

The field of indoor mobile robotics faces numerous technical, social, and economic challenges that must be addressed to achieve widespread adoption and enable truly effective human–robot collaboration [37]. These challenges span a wide spectrum, including the need for reliable navigation and mapping in dynamic indoor environments, robust and intuitive interaction modalities, adaptive behavior in response to diverse user populations, and the integration of safety, privacy, and ethical considerations. In addition, economic and market factors such as cost-effectiveness, standardization, and regulatory clarity play an important role in determining whether indoor mobile robots can be sustainably deployed across healthcare, laboratory, office, and residential settings.

### 7.1. Technical Challenges

**Figure 21:**
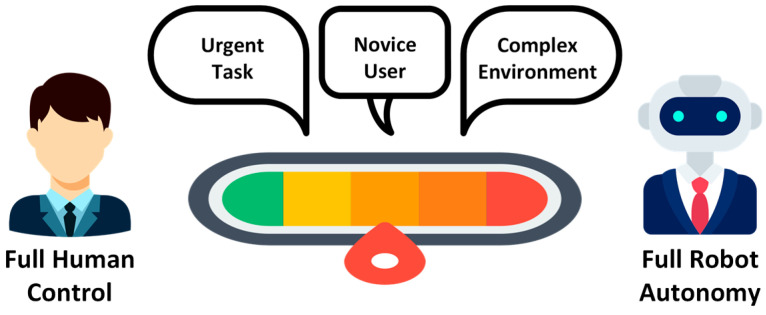
Adaptive autonomy.

**Figure 22:**
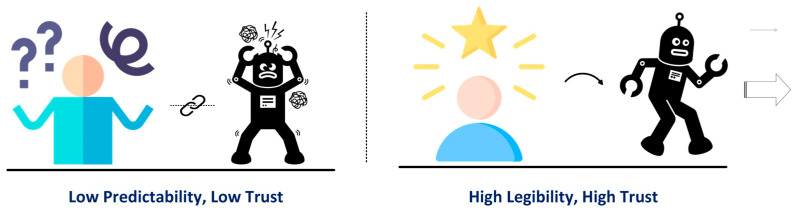
Explainable AI.

- Multi-modal Fluency: Moving beyond clunky commands to a state where robots can understand and generate communication through a seamless fusion of language, gesture, and contextual awareness, much like humans do. Speech recognition, gesture detection, and other interaction modalities must achieve near-human reliability to maintain user trust [112].
- Adaptive Autonomy: The ability of a robot to intelligently adjust its level of autonomy based on the task, the user’s expertise, and the complexity of the environment. This enables true collaboration, not just task delegation as shown in Figure 21. This requires systems that can dynamically shift between levels of autonomy, for example, from “robot decides and acts” to “human suggests, robot approves” [212].

- Explainable AI (XAI): Trust is impossible when a robot’s decision-making process is an opaque “black box”. Users need to understand the ‘why’ behind a robot’s actions, especially when those actions are unexpected. As trust is built by transparency, and hence, the reasoning for autonomous systems should be transparent and meaningful for users [199]. Figure 22 depicts this relationship of trust and predictability.

### 7.2. Social and Ethical Challenges

- Privacy and Surveillance: Indoor mobile robots raise significant privacy concerns through their sensing capabilities and data-collection practices [94]. Balancing functionality with privacy protection remains a fundamental challenge.
- Social Integration: Achieving acceptance across diverse user populations requires an understanding of cultural differences, social norms, and individual preferences [124]. Long-term social integration effects remain poorly understood.
- Trust and Reliability: Building and maintaining appropriate trust levels requires predictable robot behavior, transparent decision-making, and effective error recovery [35]. Trust calibration remains difficult, with risks of both over-trust and under-trust.
- Ethical Decision-Making: Robots operating in human environments must navigate ethical dilemmas, prioritize competing interests, and respect human autonomy [198]. Encoding ethical principles into robot behavior remains an active research area.

### 7.3. Economic and Market Challenges

- Cost–Benefit Analysis: Indoor mobile robots must demonstrate clear economic value to justify deployment costs [213]. This requires quantifying benefits such as labor savings, efficiency improvements, and quality enhancements.
- Market Fragmentation: Diverse indoor environments require specialized solutions, limiting economies of scale and increasing development costs [214]. Standardization efforts may help address this fragmentation.
- Regulatory Uncertainty: Unclear regulatory frameworks create barriers to deployment and investment [215]. Harmonized standards and clear liability frameworks are needed to support market development.
- Skills and Training: Successful robot deployment requires training for users, maintenance staff, and support personnel [14]. Educational programs and support infrastructure must develop alongside technology deployment.

### 7.4. Future Research Directions and Technology Trends

The field of indoor mobile robotics is evolving rapidly, driven by advances in AI, sensor technology, and computational infrastructure. Future research must not only address technical and social challenges but also leverage emerging technologies to develop reliable, adaptable, and human-centered robotic systems. Integrating methodological rigor into experimental studies and benchmarking new technologies will be essential to translate innovations into practical deployment.

#### 7.4.1. Future Research Directions

- Advanced AI Integration: Large Language Models (LLMs) and foundation models offer potential for more sophisticated interaction capabilities [117,127]. Driven by LLMs, robots can perceive environments, understand natural language instructions, and plan actions in a unified way [127]. Future research should focus on the safe, reliable, and ethical integration of these technologies. Methodologies may include controlled deployment studies comparing LLM-driven robots to traditional command-based systems, latency and safety benchmarking under different computational configurations (onboard vs. cloud), and simulation-based stress testing to evaluate robustness and error handling in dynamic indoor environments.
- Embodied AI: Combining perception, reasoning, and action in mobile robots requires advances in embodied intelligence and sensorimotor learning [117]. Multi-modal foundation models may enable more general-purpose capabilities. Future approaches include simulated-to-real transfer experiments to assess multi-modal sensor integration, reinforcement learning in diverse indoor layouts, and ablation studies to determine which sensor modalities most strongly contribute to task success.
- Social Learning: Robots that can learn social norms, user preferences, and environmental dynamics through observation and interaction may achieve better long-term integration [216].

Future research should include longitudinal field studies with diverse user populations to track adaptation and trust over time, employ cross-cultural evaluation protocols to assess generalizability, and implement online learning experiments to measure improvement in social behaviors.

**Figure 23:**
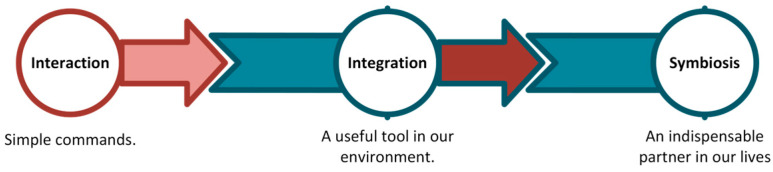
The ultimate goal: A recapitulation.

- Collaborative Intelligence: Human–robot teams that leverage complementary strengths may achieve superior performance compared to either humans or robots alone [90] as depicted in Figure 23. Research on team dynamics and coordination is needed. Methodologies may involve task-specific human–robot collaboration trials with quantitative performance metrics, the use of robot-readable environment annotations to standardize shared context, and interaction log analysis frameworks to study coordination, communication, and role adaptation. Also, for better collaboration, a robot-readable world should be the future.

- Trust and Explainable AI (XAI): Building and maintaining trust requires transparent decision-making and predictable behavior [35,199]. Future studies should integrate user-centered XAI evaluations, including think-aloud protocols and post-task questionnaires, to test the comprehension of robot reasoning, as well as performing controlled experiments varying transparency levels to determine the impact on user trust and over/under-reliance.
- Privacy, Ethics, and Regulatory Compliance: Robots must balance functionality with ethical and legal obligations [94,198]. Research should include scenario-based risk assessments for privacy and safety, simulations of regulatory-compliant operations in lab and office environments, and the development of ethics checklists and auditing protocols for real-world deployments.
- Adaptive Autonomy: Robots need to adjust autonomy levels dynamically based on task complexity and user expertise [212]. Future research should conduct hierarchical autonomy trials, where robot decision-making is systematically varied. Online learning mechanisms will be implemented to tune autonomy levels in real-time. An important part is also the evaluation of human–robot performance using task success rates and cognitive load metrics.

#### 7.4.2. Technology Trends and Opportunities

Emerging technologies and infrastructure developments provide new opportunities to enhance the capabilities, reliability, and affordability of indoor mobile robots. Exploiting these trends effectively will require rigorous testing, benchmarking, and integration strategies to ensure that innovations translate into real-world improvements.

- 5G and Edge Computing: High-bandwidth, low-latency communications enable cloud-based AI processing while maintaining real-time responsiveness [101]. This may enable more sophisticated capabilities in resource-constrained mobile platforms. Future research should evaluate task performance, latency, and reliability under different network configurations, including hybrid edge-cloud setups, to determine optimal architectures for resource-constrained mobile platforms.
- Sensor Miniaturization: Advances in sensor technology enable richer environmental perception in smaller, less expensive packages [102]. This trend may enable new interaction modalities and improved system performance. Methodologies such as ablation studies, sensor fusion benchmarking, and comparative trials across diverse indoor layouts can identify which sensor combinations most effectively support navigation, interaction, and safety.
- Battery Technology: Improvements in energy density and charging speed may enable longer operation times and reduced downtime [214]. Research should explore wireless charging, energy harvesting, and adaptive power-management strategies through long-duration field trials to quantify real-world gains in operational efficiency.
- Manufacturing Advances: 3D printing, modular design, and automated assembly may reduce costs and enable customization for specific applications [187]. These advances may help address market fragmentation challenges. An evaluation of modular and scalable manufacturing approaches through prototyping and a cost–benefit analysis will help address market fragmentation and support broader deployment.

These future research directions and emerging technological trends provide a roadmap for advancing indoor mobile robotics. By combining methodological rigor, AI-driven capabilities, adaptive autonomy, and robust sensor and infrastructure technologies, researchers and practitioners can move toward more reliable, socially aware, and economically viable robotic systems. The following conclusions summarize the key insights and implications of these developments for human–robot collaboration in diverse indoor environments.

## 8. Conclusions

This comprehensive review of Human–Robot Interaction in indoor mobile robotics reveals a rapidly evolving field with significant potential for transforming how humans and robots collaborate in shared environments. The analysis of interaction modalities demonstrates that effective HRI requires multimodal approaches that combine speech, gesture, touch, and visual communication channels. Each modality offers unique advantages while facing distinct challenges related to environmental conditions, user diversity, and technical limitations.

The examination of user experience and acceptance factors highlights the critical importance of human-centered design in achieving successful robot deployment. Trust, reliability, and social integration emerge as fundamental requirements that must be addressed through careful attention to robot behavior, transparency, and cultural sensitivity.

The case studies of Moxi, Temi, and Astro illustrate different approaches to addressing these challenges, with varying degrees of success depending on the application domain and user requirements.

Safety and privacy considerations present ongoing challenges that require continued attention from researchers, manufacturers, and policymakers. The development of appropriate regulatory frameworks, technical standards, and ethical guidelines will be essential for the widespread adoption of indoor mobile robots. Privacy protection mechanisms and transparent data practices must be integrated into system design rather than treated as afterthoughts.

The technical challenges identified in this review, including navigation reliability, interaction robustness, and computational constraints, represent active areas of research with promising solutions emerging from advances in AI, sensor technology, and computing infrastructure. The integration of Large Language Models and foundation models offers promise for enhancing interaction capabilities while raising new questions about safety and reliability.

Future research should focus on developing more robust and adaptable interaction systems that can operate effectively across diverse indoor environments and user populations. Long-term studies of human–robot relationships, social integration effects, and trust development will be crucial for understanding the full implications of robot deployment in human-centered environments.

The success of indoor mobile robotics will ultimately depend on achieving the right balance among technical capability, user acceptance, safety, and economic viability. This requires continued collaboration among technologists, social scientists, ethicists, and end users to ensure that robotic systems truly serve human needs and values. As the field matures, the focus must shift from demonstrating technical feasibility to creating sustainable, beneficial, and trustworthy partnerships between humans and robots in our most personal and important spaces.

## Figures and Tables

**Table 1:** Comparison of robotic operational contexts.

Context	Interaction Priorities	Operational Constraints	Notes
Structured & Predictable	Unwavering efficiency and precision	Repetitive and pre- programmed tasks	Limited and highly controlled human interaction; static environment
Unstructured & Dynamic	Safe, adaptive, and socially intelligent assistance	Varied and context- dependent tasks	Continuous interaction with untrained users (adults, children, pets); dynamic clutter

**Table 2:** Diverse user groups for Human–Robot Interactions.

Context	Interaction Priorities	Operational Constraints	Notes
Healthcare	Hands-free, reliability, clarity, efficiency	Sterility, shift-length runtime	Elevator, door integration
Laboratory	Precision, traceability, zero-contact interaction	Safety, contamination control	Delicate equipment handling
Office	Low-friction tasking, efficiency	BYOD networks, mixed spaces	Meeting etiquette, proxemics
Home	Approachability, privacy, trust, emotional intelligence	Diverse users, clutter	Family routines, consent

**Table 3:** Comparative overview of domain-specific requirements for indoor mobile robots.

Domain	Primary Objective	Interaction Focus	Environmental Structure
Healthcare	Workflow relief, patient support	Clear, reliable, emotionally sensitive	Semi-structured, high traffic
Laboratory	Precision workflow automation	Predictable, concise communication	Highly structured
Office	Low-friction service support	Socially appropriate, non-disruptive	Structured, but socially dynamic
Industrial	Productivity and safety	Task-oriented, multimodal command	Structured, safety regulated
Residential	Assistance and companionship	Adaptive, personalized, privacy-aware	Unstructured, dynamic

**Table 4:** Domain comparison.

Domain	Structure	Technical Rigidity	Social Complexity	Dominant HRI Requirements
Industrial	Highly structured	High	Low	Predictable interaction, safety compliance
Laboratory	Highly structured	Very high	Moderate	Safety, intent signaling
Healthcare	hybrid	High	High	Trust, clarity, contextual awareness
Office	Semi-structured	Moderate	Moderate–high	Etiquette awareness, proxemics
Residential	Highly dynamic	Moderate	Very high	Emotional intelligence, trust-building

**Table 5:** Approaches to social navigation in robotics.

Context	Interaction Priorities	Operational Constraints
Reactive	Immediate obstacle avoidance	Simple rule-based logic
Predictive	Proactive path planning	Anticipation of human trajectories
Model-based	Adherence to proxemics	Explicit social rule implementation
Learning-based	Socially compliant behaviors	Reinforcement learning and simulation

**Table 6:** Overview of speech recognition approaches for indoor mobile robots.

	Keyword-Based AST	Command + Grammar NLP	Template-Based/Rule-Based	Statistical/N-Gram Models
Typical Datasets/Test Environments	Small vocabulary sets, lab-controlled speech	Structures tasks, simulated indoor environments	Predefined task environments	Controlled speech corpora
Evaluation Metrics	Accuracy, latency	Accuracy, task success rate	Accuracy, user completion	Perplexity, accuracy
Strengths/Advantages	Low computational cost, simple deployment	Moderate flexibility, handles simple context	Deterministic behavior, predictable	Can model probabilistic commands
Limitations/Gaps	Limited vocabulary, poor context understanding, noise sensitive	Medium cognitive load, limited adaptability	Rigid, fails under unexpected inputs	Limited semantic understanding, poor generalization
References	[113,114,115]	[116,118]	[123]	[112]

**Table 7:** Comparative analysis of LLM- and VLM approaches for speech-based HRI.

	GPT-Style LLM (Onboard)	LLM + Cloud Inference	VLM-Enhanced Speech + Vision	Transformer-Based Multimodal Fusion
Typical Datasets/Test Environments	Domain-specific corpora, interactive tasks	Open-domain dialogues, multimodal indoor datasets	Indoor navigation + multimodal interaction datasets	Simulated and real-world interactive tasks
Evaluation Metrics	Task success, command comprehension, latency	Task success, latency, robustness, user satisfaction	Task success, robustness, user satisfaction	Task completion, cognitive load, robustness
Strengths/Advantages	Context-aware reasoning, flexible dialogue, supports complex instructions	Adaptive, context-aware, can integrate multimodal input streams	Integrates visual context for disambiguation, improves sematic understanding	Combines speech and gestures, adapts to user preferences and environmental conditions
Limitations/Gaps	High computational cost, memory-intensive, limited onboard resources	Latency, network dependency, privacy concerns, reliability in critical tasks	High computational cost, sensor calibration required, safety-critical constraints	Complex fusion algorithms, temporal synchronization issues, resource-heavy
References	[126]	[127]	[128]	[129,130]

**Table 8:** Overview comparison of speech-based HRI approaches.

Criterion	Classical Speech Methods	LLM/VLM-Based Methods
Approach/Models	Keyword-based AST, Command Grammar + NLP	GPT-style LLM (onboard), LLM + cloud inference, VLM-enhanced fusion
Computational Cost	Low–medium	Medium–high
Robustness to Noise	medium	high
User Cognitive Load	Low–medium	low
Hardware Requirements	Microphone, CPU	GPU, Camera, Microphone, network/CPU
Typical Test Environment/Datasets	Lab-controlled speech, small vocabulary sets	Interactive indoor tasks, multimodal datasets
Evaluation Metrics	Accuracy, latency, task completion	Task success, comprehension, robustness, user satisfaction
Strengths/Advantages	Simple, predictable, low-cost	Context-aware, flexible, multimodal, adaptive
Limitations/Gaps	Limited vocabulary, poor context understanding	High computational cost, latency, network dependency, safety-critical concerns
References	[113,114,115,116,118,123]	[126,127,128,130,131,132]

**Table 9:** Summary of Gesture-Based HRI Methods, Evaluation Metrics, and Limitations.

	Template/Rule-Based	ML-Based Classifiers	Deep Learning (LSTM, Transformers)
Typical Environments/Data sets	Lab-controlled, predefined gestures	Gesture datasets, indoor tasks	Simulated + real-world interactive tasks
Evaluation Metrics	Accuracy, task completion	Accuracy, F1-score, latency	Task success, recognition rate, latency
Strength/Advantages	Simple, low computational cost, predictable	Adaptive to user-specific gestures	Capture sequential and subtle cues, real-time
Limitations/Gaps	Rigid, limited gestures, poor generalization	Requires labeled data, moderate computational cost	High computational cost, requires sensor calibration
References	[138,140]	[139,141]	[136,137,142,143,144]

**Table 10:** Summary of touch and physical interaction HRI methods, evaluation metrics, and limitations.

	Touchscreen Interfaces	Physical Guidance	Haptic Feedback	Biometric Access
Typical Technologies	Capacitive screens, GUI systems	Kinesthetic teaching, force sensors	Vibrotactile motors, force feedback	Fingerprint sensors
Evaluation Metrics	Task completion time, error rate, usability scores	Position accuracy, response time, safety compliance	Reaction time, user perception accuracy	Authentication accuracy, latency
Advantages	Familiar, high information density	High precision, intuitive correction	Accessibility support, confirmation feedback	Secure access control
Limitations	Visibility issues, mobility constraints	Requires proximity, safety risks	Limited bandwidth, hardware complexity	Sensor reliability, hygiene concerns

**Table 11:** Summary of visual and gaze-based HRI methods: Approaches, metrics, and limitations.

	Gaze Tracking	Facial Expression Recognition	Face Recognition/Biometric	Visual Feedback Displays
Typical Technologies	Eye trackers, camera-based algorithms	RGB cameras, CNNs, DL models	RGB/IR camera, face embeddings	LEDs, screens, expressive robot motion
Evaluation Metrics	Accuracy of gaze detection, latency, task performance	Recognition accuracy, response time, affect detection	Authentication accuracy, latency	User comprehension, task efficiency
Advantages	Attention-aware behavior, spatial referencing	Infers emotional state, supports adaptive behavior	Secure access, personalized interaction	Communicates robot intent, social cues
Limitations	Sensitive to lighting, occlusion and head movement	Variability in expressions, cultural differences, computational cost	Privacy concern, sensor reliability	Limited expressiveness, interpretation varies by user

**Table 12:** Interaction modalities in indoor mobile robotics.

Modality	Advantages	Challenges	Best Applications	User Accessibility
speech	natural, hands-free, multilingual support	noise sensitivity; privacy concerns; multispeaker confusion	healthcare; domestic assistance	limited for hearing-impaired users
Gesture	intuitive; conveys spatial information; often culturally universal	lighting dependent; occlusion issues; limited vocabulary; training required	navigation; object manipulation	limited for mobility-impaired users
Touch	direct feedback; precise control; familiar UI	safety risks; requires proximity	emergency stops; detailed configuration	high accessibility
Visual/Gaze	social cues; attention awareness; nonintrusive	privacy concerns; lighting-sensitive	social interaction; attention management	limited for visually impaired users
Multimodal	robust; adaptive; comprehensive	complex; computationally intensive	complex tasks across domain	highest accessibility

**Table 13:** Multimodal/VLM interaction.

Modality	Gesture + Speech	Multimodal + LLM	VLM-Based Perception
Approach/Model	Classical Model	Transformer-based fusion	CLIP * or similar
Computational Cost	Medium	High	Medium to high
Robustness to Noise	Medium	High	high
User Cognitive Load	Medium	Low	low
Hardware Requirements	Camera + microphone	GPU + Camera + microphone	GPU + camera
References	[134,140,155]	[117,127]	[129,132]

* CLIP: Contrastive Language-Image Pretraining (OpenAI model).

**Table 14:** Comparative analysis of interaction modalities in indoor mobile robotics.

	Speech	Gesture	Touch/Physical	Visual/Gaze	Multimodal + LLM/VLM
Approach/Model	Keyword-based AST; Command Grammar + NLP	Template-based; ML-based; Deep Learning (LSTM/Transformer)	Touchscreens, force-sensitive surfaces, haptic feedback	Eye tracking, facial expression, body pose, proxemics	Fusion of speech, gesture, touch, visual inputs; Transformer-based or CLIP-like models
Computational Cost	Low–Medium	Medium	Low–medium	Medium–high	High
Robustness to Noise/Environment	Medium; sensitive to ambient noise	Medium; lighting/occlusion sensitive	High, proximity required	Medium; lighting, movement sensitive	High
User Cognitive Load	Low–Medium	Medium	Low	Medium	Low
Accessibility	Limited for hearing-impaired users	Limited for mobility-impaired users	High accessibility	Limited for visually impaired users	High
Typical Applications	Healthcare, domestic assistance	Navigation, object manipulation, collaborative tasks	Direct robot control, configuration, emergency interventions	Social interaction, attention management, secure access	Complex tasks, adaptive interaction, context-aware operation
References	[112,114,116,118]	[133,136,139,142]	[145,146,147,148]	[149,150,151,152]	[127,129,132,153,155]

**Table 15:** Translation of human–human trust factors to human–robot trust paradigms.

Human-Human Trust	Basis	Human-Robot Trust	Translation
Dependability	Reliability of intentions and actions	Transparency	System explainability; lack of transparency negatively impacts trust
Generosity	Caring for others’ well-being	Predictability	Anticipation of regular behavior that meets user requirements
Competence	The ability to perform tasks effectively.	Autonomy and Flexibility	Robot’s ability to accomplish tasks efficiently
Sincerity	Honesty, fairness, and integrity	Emotional Engagement	Perceived warmth and empathy of the robot

**Table 16:** Comparative analysis of Moxi, Temo, and Astro.

Dimension	Moxi (Diligent Robotics)	Temi	Amazon Astro
Primary domain	Healthcare (hospital logistics)	Home and office personal assistance	Home security and assistance
Target users	Nurses, hospital staff	Families, elderly users, offices	Families, elderly individuals, smart-home users
Core value proposition	Task automation (supply delivery) to free clinical staff	Voice-controlled mobile assistant for communication and entertainment	Mobile home monitoring and ecosystem integration
Interaction design	Multimodal (speech, gesture, LED gaze, audio cues)	Voice-first, touchscreen, mobile app	Voice, visual expressions, ambient sensing
Navigation and mobility	Advances hospital SLAM, elevator and badge-access integration	SLAM-based navigation, follow-me mode	Advances SLAM, autonomous patrol routines
Manipulation capability	Yes (mobile manipulation for delivery tasks)	No physical manipulation	No manipulation (monitoring-focused mobility)
Autonomy level	High (infrastructure integration, remote LTE monitoring)	Moderate (navigation autonomy, limited physical interaction)	High navigation autonomy, ecosystem-dependent functionality
Predictability	Very high (routine routes, gaze-direction cues, structures error loops)	Moderate (less explicit signaling of system state)	High for patrol routines and scheduled behaviors
Transparency mechanisms	Explicit error messages, gaze cues, audio alerts, remote developer loop	Limited explainability of always-listening cloud processing	Strong hardware cues (green/blue lights), physical privacy controls
Emotional engagement	Subtle anthropomorphism (animated eyes, gratitude behaviors)	Conversational warmth, adaptive screen positioning	Animal-like digital eyes, companion-style behavior
Privacy implications	Moderate (clinical data integration, regulated environment)	Significant (cloud-based voice and facial recognition)	High (continuous monitoring, ecosystem-wide data processing)
Infrastructure integration	Deep integration (Hospital IT, elevators, badge system)	Smart home integration, cloud AI services	Extensive amazon ecosystems and Ring integration
Major strength	Domain-specific reliability and workflow integration	Accessibility and ease of use	Ecosystem integration, hardware transparency
Primary limitations	Complex hospital integration, explainability gaps in failure cases	Limited task range (no manipulation), privacy concerns	Privacy concerns, high cost, ecosystem lock-in
HRI design philosophy	Human-aware professional assistant	Accessible consumer companion	Mobile smart-home security platform

## Data Availability

No new data were created or analyzed in this study. Data sharing is not applicable to this article.

## Abbreviations

The following abbreviations are used in this manuscript:

AGV	Automated Guided Vehicle
AI	Artificial Intelligence
AMR	Autonomous Mobile Robot
API	Application Programming Interface
ASR	Automatic Speech Recognition
BCI	Brain–computer interface
CLIP	Contrastive Language-Image Pretraining
CPU	Central Processing Unit
DL	Deep Learning
GDPR	General Data Protection Regulation
GenAI	Generative Artificial Intelligence
GPT	Generative Pre-trained Transformer
GPU	Graphics Processing Unit
GUI	Graphical User Interface
HRC	Human–Robot Collaboration
HRI	Human–Robot Interaction
ICU	Intensive Care Unit
IMU	Inertial Measurement Unit
IR	Infrared
LBM	Large Behavioral Model
LiDAR	Light Detection and Ranging
LLM	Large Language Model
LSTM	Long Short-Term Memory
LVQ	Learning Vector Quantization
NLP	Natural Language Processing
PPE	Personal Protective Equipment
PRISMA	Preferred Reporting Items for Systematic Reviews and Meta-Analyses
RGB	Red-Green-Blue
RGB-D	Red-Green-Blue plus Depth
SLAM	Simultaneous Localization and Mapping
SVM	Support Vector Machine
TAM	Technology Acceptance Model
TTS	Text-to-Speech
UI	User Interface
UWB	Ultra-Wideband
UX	User Experience
VLM	Vision-Language Model
XAI	Explainable Artificial Intelligence
